# Characterization of *P. falciparum* dipeptidyl aminopeptidase 3 specificity identifies differences in amino acid preferences between peptide‐based substrates and covalent inhibitors

**DOI:** 10.1111/febs.14953

**Published:** 2019-06-24

**Authors:** Laura E. de Vries, Mateo I. Sanchez, Katarzyna Groborz, Laurie Kuppens, Marcin Poreba, Christine Lehmann, Neysa Nevins, Chrislaine Withers‐Martinez, David J. Hirst, Fang Yuan, Shirin Arastu‐Kapur, Martin Horn, Michael Mares, Matthew Bogyo, Marcin Drag, Edgar Deu

**Affiliations:** ^1^ Department of Medical Microbiology Radboud University Medical Center Nijmegen The Netherlands; ^2^ Department of Genetics Stanford School of Medicine Stanford CA USA; ^3^ Division of Bioorganic Chemistry Faculty of Chemistry Wroclaw University of Technology Wroclaw Poland; ^4^ Chemical Biology Approaches to Malaria Laboratory The Francis Crick Institute London UK; ^5^ Computational Sciences GlaxoSmithKline Collegeville PA USA; ^6^ Malaria Biochemistry The Francis Crick Institute London UK; ^7^ Crick‐GSK Biomedical LinkLabs GlaxoSmithKline Stevenage UK; ^8^ Department of Pathology Stanford University School of Medicine Stanford CA USA; ^9^ Institute of Organic Chemistry and Biochemistry Czech Academy of Sciences Prague Czech Republic

**Keywords:** dipeptidyl aminopeptidase, malaria, positional scanning, proteases, specificity

## Abstract

Malarial dipeptidyl aminopeptidases (DPAPs) are cysteine proteases important for parasite development thus making them attractive drug targets. In order to develop inhibitors specific to the parasite enzymes, it is necessary to map the determinants of substrate specificity of the parasite enzymes and its mammalian homologue cathepsin C (CatC). Here, we screened peptide‐based libraries of substrates and covalent inhibitors to characterize the differences in specificity between parasite DPAPs and CatC, and used this information to develop highly selective DPAP1 and DPAP3 inhibitors. Interestingly, while the primary amino acid specificity of a protease is often used to develop potent inhibitors, we show that equally potent and highly specific inhibitors can be developed based on the sequences of nonoptimal peptide substrates. Finally, our homology modelling and docking studies provide potential structural explanations of the differences in specificity between DPAP1, DPAP3, and CatC, and between substrates and inhibitors in the case of DPAP3. Overall, this study illustrates that focusing the development of protease inhibitors solely on substrate specificity might overlook important structural features that can be exploited to develop highly potent and selective compounds.

## Graphical abstract

We have used peptide‐based libraries of substrates and covalent inhibitors to characterize the specificity of *Plasmodium* dipeptidyl aminopeptidase 3, a protease important for red blood cell invasion. Interestingly, we observed significant differences in amino acid preference between substrates and inhibitors, and showed that highly potent and specific inhibitors can be developed based on the sequences of nonoptimal peptide substrates.

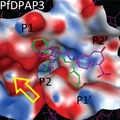

**Abbreviations**
1Nal: 1‐naphthalene
2fa: 2‐furylalanine
2Nal: 2‐naphthalene
2ta: 2‐thiofurylalanine
3Abz: 3‐amino‐benzoic acid
AA: amino acid
ABP: activity‐based probe
ACC: 7‐amino‐4‐carbamoylmethylcoumarin
Acpc: 1‐aminocyclopropanecarboxylic acid
Aib: 2‐aminoisobutiric acid
Ala(NH_2_): aminoalanine
Amb: amino‐1‐methyl‐benzyl
AMC: 7‐amino‐4‐methylcoumarin
Amc: amino‐1‐methyl‐cyclohexyl
Arg(NO_2_): nitroarginine
Bip: biphenylalanine
Bpa: 4‐benzoyl‐phenylalanine
Cat: cathepsin
Cba: 2‐amino‐4‐cyanobutyric acid
Cha: cyclohexylalanine
Chg: cyclohexylglycine
Cit: citruline
DPAP: dipeptidyl aminopeptidase
FP: falcipain
Glu(Bzl): glutamic acid benzyl ester
hAla(Bht): (4‐benzothiazol‐2‐yl)homoalanine
hAla(NH_2_): aminohomoalanine
hAla: homoloanine
hArg: homoarginine
hPG: homoprolylglycine
hPhe: homophenylalanine
hPro: homoproline
hSer(Bzl): homoserine‐*O*‐benzyl
hSer: homoserine
Igl: indanyl‐glycine
Inp: piperidine‐4‐carboxylic acid
iRBC: infected red blood cell
KO: knockout
nLeu(o‐Bzl): 6‐benzoyloxynorleucine
nLeu: norleucine
nVal: norvaline
Orn: ornithine
Phe(Guan): 4‐guanidino‐phenylalanine
Phe(I): 4‐iodophenylalanine
Phe(Me): 4‐methyl‐phenylalanine
Phe(NH_2_): 4‐aminophenylalanine
Phg: phenylglycine
PS‐SCL: positional scanning synthetic combinatorial libraries
RBC: red blood cell
SAR: structure‐activity relationship
tLeu: tert‐leucine
Tyr(Bzl): tyrosine‐*O‐*benzoyl
Tyr(NO_2_): 3‐nitrotyrosine
VS: vinyl sulfone
βNA: beta‐naphthylamide

## Introduction

Malaria is a devastating infectious parasitic disease causing nearly half a million deaths every year 1. Malaria is caused by parasites of the *Plasmodium* genus and is transmitted by *Anopheles* mosquitoes during a blood meal. Within the mosquito midgut, parasites reproduce sexually, multiply and travel to the salivary glands from where they are transmitted to the human host. Upon infection, parasites first establish an asymptomatic infection in the liver, followed by exponential asexual replication in the blood stream through multiple rounds of red blood cell (RBC) invasion, intracellular replication and egress from infected RBCs. This erythrocytic cycle is responsible for the symptoms and pathology of malaria. Over the last 15 years, the world has seen a very significant drop in malaria incidence, mainly due to the global distribution of insecticide‐impregnated bed nets and the use of artemisinin‐based combination therapies as the standard of care for uncomplicated malaria 2. However, malaria remains a major global health burden with half of the world population at risk and around 200 million clinical cases per year. Unfortunately, mosquitoes are becoming increasingly resistant to insecticides 3, and artemisinin resistance is on the rise 4, thus making the identification of antimalarial targets and the development of drugs with novel mechanisms of action are extremely urgent 5.

Dipeptidyl aminopeptidases (DPAPs) are papain‐fold cysteine proteases that are expressed at all stages of parasite development 6, 7 and might therefore be viable drug targets to treat malaria and prevent its transmission. DPAPs recognize the free N‐terminus of protein substrates and cleave N‐terminal dipeptides 8, 9. The mammalian homologue cathepsin C (CatC) is the best studied DPAP 10. In most cells, CatC plays a catabolic lysosomal function. However, in immune cells it is responsible for activating various granule serine proteases involved in the immune response and inflammation such as neutrophil elastase, chymase, granzyme A and B or cathepsin G 11, 12, 13, 14. Because of its role in activating pro‐inflammatory proteases, CatC has been pursued as a potential target for chronic inflammatory diseases 15, 16, 17. Phase I clinical trials with CatC inhibitors have been performed by GSK (GSK2793660) 18 and AstraZeneca (AZD7986) 19, thus proving that DPAPs can be targeted with small drug‐like molecules.

Three DPAPs are conserved across *Plasmodium* species but very little is known about their molecular functions. In *P. falciparum*, the most virulent *Plasmodium* species responsible for 90% of malaria mortality, attempts to directly knockout (KO) DPAP1 20 or DPAP3 21 have been unsuccessful, suggesting that they are important for parasite replication. In the *P. berghei* murine model of malaria, KO of DPAP1 or DPAP3 results in a significant decrease in parasite replication 22, 23, 24. DPAP1 localizes mainly in the digestive vacuole 20, an acidic organelle where degradation of haemoglobin takes place. This proteolytic pathway provides a source of amino acids for protein synthesis and liberates space within the RBC for parasites to grow. DPAP1 has been proposed to play an essential role at the bottom of this catabolic pathway 20, 25. However, this function has not yet been confirmed genetically. Previously published inhibition studies suggested that DPAP3 was at the top of the proteolytic cascade that controls parasite egress form iRBCs 26. However, our recent conditional KO studies have disproven this hypothesis and shown instead that DPAP3 activity is critical for efficient RBC invasion 21. Finally, DPAP2 is only expressed in sexual stages and has been shown to be important for gamete egress from iRBCs, thus making it a potential target to block malaria transmission 27, 28. Overall, a pan‐DPAP inhibitor will target the parasite at different stages of development, thus potentially slowing down the emergence of resistance.

A clear understanding of the determinants of substrate specificity of *Plasmodium* DPAPs and CatC will be required in order to develop pan‐DPAP inhibitors with minimal off‐target effects on host CatC, and to design highly specific inhibitors to study the biological function of DPAP1 and DPAP3. In this article we will use the accepted Schechter and Berger nomenclature to describe the specificity of proteases 29. Residues upstream of the scissile bond will be referred to as P1, P2, P3, etc. Their side chains bind into the S1, S2, S3, etc pockets of the active site respectively. Residues downstream of the scissile bond are referred to as P1′, P2′, P3′, etc, and they bind into the corresponding S1′, S2′, S3′, etc pockets. The scissile bond is between the P1 and P1′ positions.

A general approach to determine the specificity of proteases upstream of the scissile bond (nonprime pockets) is the use of positional scanning substrate libraries where a fluorophore is conjugated to the C terminus of a peptide library via an amide bond. Proteolytic cleavage of this bond results in a significant increase in fluorescence intensity allowing accurate measurement of substrate turnover. The most common libraries used for this purpose are positional scanning synthetic combinatorial libraries (PS‐SCL) 30, 31, 32. PS‐SCL are composed of multiple sub‐libraries designed to determine the specificity of each nonprime binding pocket in a protease. In each sub‐library, the amino acid (AA) at a specific position is varied while a stoichiometric mixture of all natural AAs is used in all other positions. PS‐SCL thus provide the substrate specificity at each site in the context of all possible combination of AAs at all other positions. Alternatively, the specificity of a given binding pocket can be determined by varying the identity of the AA at that position while fixing the rest of the peptide to residues known to be recognized by the protease of interest. This approach has been used to fingerprint the specificity of amino exopeptidases such as aminopeptidases 33, 34 or DPAPs 35, which only recognize one or two AAs upstream of the scissile bond respectively. PS‐SCL have also been applied to protease inhibitor libraries by replacing the fluorophore with a reversible or irreversible warhead 36. Optimum substrates and inhibitors are then designed by combining the best residues in each position. Importantly, the recent incorporation of non‐natural AAs into these libraries has significantly increased the chemical space that can be explored to characterize the specificity of proteases and has allowed the design of substrates and inhibitors with enhanced selectivity over compounds that contain only natural amino acids 37, 38, 39, 40.

Structure–activity relationship (SAR) studies with positional scanning substrate and inhibitor libraries have been performed both on DPAP1 and CatC 25, 35, 41, but to a much lesser extent on DPAP3 26. Here, we used libraries of peptide‐based substrates and inhibitors to determine the specificity of *P. falciparum* DPAP3 at the P1 and P2 positions. Importantly, the libraries used in this study have been previously screened against DPAP1 and CatC and are therefore ideal to compare the specificities of these three proteases 35. Our studies show that DPAP3 preferentially cleaves after basic and large aromatic residues (P1 position), and that it prefers substrates having N‐terminal aliphatic residues (P2 position). We also identified several non‐natural P2 residues that are exclusively recognized by either DPAP1 or DPAP3. By combining the SAR information obtained from these substrate and inhibitor screens, we developed specific DPAP1 and DPAP3 inhibitors that remain selective in live parasites. Interestingly, while SAR information obtained from positional scanning substrate libraries is often used to develop potent protease inhibitors 39, we have identified significant differences in specificity between substrates and inhibitors, particularly in the case of DPAP3. Homology modelling and docking studies provide structural explanations about the differences in specificity between these enzymes, and between substrates and inhibitors in the case of DPAP3. Overall, our study shows that while highly potent inhibitors can be designed based on the sequence of optimal substrates, equally potent and specific inhibitors can be developed using sequences of nonoptimal substrates.

## Results

### DPAP3 substrate specificity

**Figure 1:**
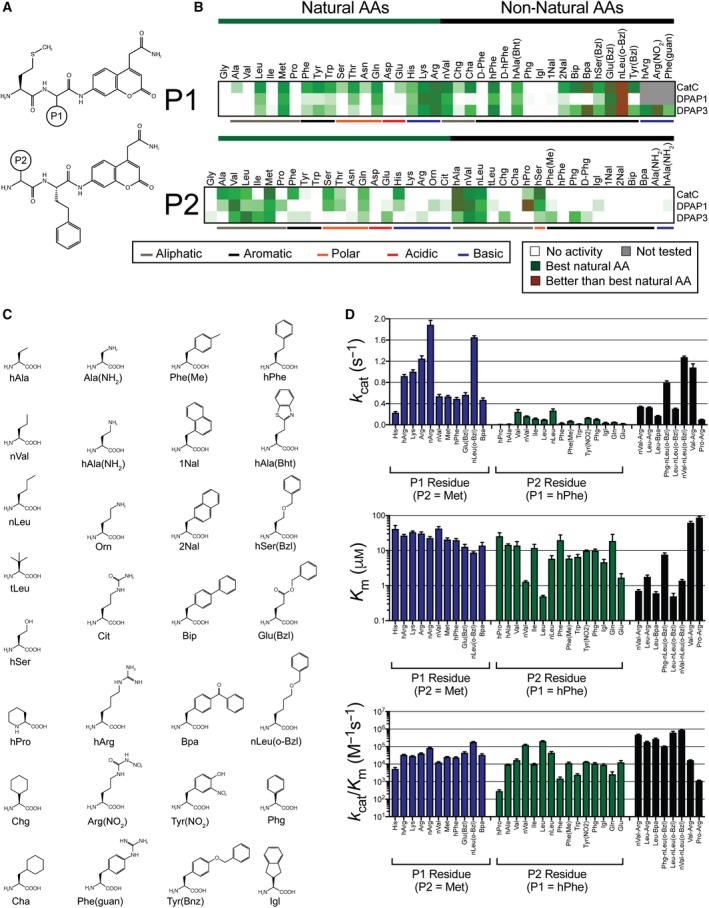
DPAP3 substrate specificity. (A) Structure of P1 and P2 substrate libraries. (B) Heat map comparing relative turnover rates for the different DPAPs at 1 μm substrate. For each enzyme and substrate, turnover rates were normalized relative to the best natural AA (dark green): Arg in P1 for all DPAPs; Met, Val and Leu in P2 for CatC, DPAP1, and DPAP3 respectively. Red indicates substrates that are turned over better than the best natural AA. White represents no activity, and grey substrates that were only tested on DPAP3. (C) Structure of non‐natural AAs used in the substrate library. (D) Steady‐state Michaelis–Menten parameters for DPAP3 determined for selected substrates. Error bars represent the standard deviation of each parameter (*N* = 3‐10 depending on the substrate, see Table 1).

A positional scanning library of 96 substrates (Fig. 1A), composed of a P1 sub‐library of 39 substrates (P2 fixed to Met) and a P2 sub‐library of 57 substrates (P1 fixed to homophenylalanine (hPhe)), was screened at 1 μm against recombinant DPAP3 (DPAP3; Fig. 1B,C). The fixed P1 and P2 side chains were selected based on previously known AA preferences for CatC. The heat map shown in Fig. 1B compares the specificities of DPAP3 with those previously obtained for DPAP1 and CatC 35 at the same substrate concentration. Note that *D*‐Phg is the only *D‐*AA in P2 that is cleaved by DPAP3, albeit poorly. The remaining 17 substrates containing *D‐*AAs in P2 (*D‐*hPhe and all natural *D‐*AAs except *D‐*Cys and *D*‐Met) were not cleaved by DPAP3, nor by DPAP1 or CatC. To simplify Fig. 1, D*‐*Phg is the only substrate containing a *D‐*AA that is shown.

All three DPAPs show broad and similar P1 specificity, which is not surprising because in clan CA proteases the P1 residue side chain is solvent‐exposed. For all DPAPs, a general preference for long basic, aliphatic and aromatic P1 residues was observed—basic: Lys, Arg, homoarginine (hArg), and nitroarginine (Arg(NO_2_)); aliphatic: Met, norvaline (nVal) and Leu; aromatic: hPhe, (4‐benzothiazol‐2‐yl)homoalanine (hAla(Bht)), 6‐benzyloxynorleucine (nLeu(o‐Bzl)), glutamic acid benzyl ester (Glu(Bzl)) and homoserine‐*O*‐benzyl (hSer(Bzl))—. Interestingly, DPAP1 differs from CatC and DPAP3 in that it does not accept large hydrophobic groups such as cyclohexylalanine (Cha), 2‐naphthalene (2Nal), biphenylalanine (Bip), 4‐benzoyl‐phenylalanine (Bpa), tyrosine‐*O*‐benzoyl (Tyr(Bzl)), or 4‐guanidino‐phenylalanine (Phe(Guan)) in P1. Unfortunately, we could not identify any P1 residue that was recognized by DPAP1 and/or DPAP3 but not by CatC.

Clear differences in specificity were observed between the three DPAPs at the P2 position (Fig. 1B). DPAP3 seems to have a narrower P2 specificity for natural AAs than DPAP1 or CatC, probably reflecting its more specific biological function in RBC invasion compared to the catabolic functions of DPAP1 and CatC. Both DPAP1 and DPAP3 have a strong preference for long aliphatic residues such as Leu, Ile, norleucine (nLeu), Met or norvaline (nVal). Some non‐natural AAs seem to be exclusively cleaved by DPAP3 such as cyclohexylglycine (Chg), Phg or 4‐methyl‐phenylalanine (Phe(Me)). Surprisingly, Phe(Me) is the only substrate in the library with an aromatic P2 residue that is accepted by DPAP3 even though vinyl sulfone (VS) inhibitors with P2 aromatic residues such as Tyr, Trp or nitrotyrosine (Tyr(NO_2_)) have been shown to be potent DPAP3 inhibitors 21, 26. Interestingly, an Ile in P2 is efficiently cleaved by both *Plasmodium* DPAPs but not by CatC.

### Development of optimum DPAP3 substrates

To determine how P1 and P2 side chains influence *k*_cat_ and *K*_m_ for DPAP3, we performed Michaelis–Menten studies on selected substrates from the P1 and P2 libraries. In addition, we synthesized a series of substrates that combine optimal natural and non‐natural AAs for DPAP3: Arg, hPhe, nLeu(o‐Bzl) and Bpa in P1, and Leu, Val, nVal and Phg in P2. We also tested substrates predicted to be DPAP1‐selective (Pro‐Arg‐ACC and hPro‐hPhe‐ACC) against DPAP3. Finally, because we were surprised by the lack of activity observed for substrates with aromatic P2 residues, we measured Michaelis–Menten parameters for Phe‐Arg‐ACC, Trp‐hPhe‐ACC, and Tyr(NO_2_)‐hPhe‐ACC. The sequence of the last substrate is based on the structure of SAK1 (Tyr(NO_2_)‐hPhe‐VS), which is the most potent DPAP3 inhibitor identified so far (see below). Table 1 and Fig. 1D report the Michaelis–Menten parameters determined for all these substrates (Michaelis–Menten curves are shown in Fig. S1).

**Table 1:** Steady‐state Michaelis–Menten parameters for DPAP3

Substrate	*N*	*k*_cat_ (s^−1^)	*K*_m_ (μm)	*k*_cat_/*K*_m_ ·10^−3^ (m ^−1^·s^−1^)
Met‐His‐ACC	4	0.23 ± 0.02	42 ± 10	5.4 ± 1
Met‐nVal‐ACC	4	0.54 ± 0.04	43 ± 5	13 ± 1
Met‐hPhe‐ACC	4	0.5 ± 0.02	20 ± 2	24 ± 2
Met‐Met‐ACC	4	0.54 ± 0.02	21 ± 2	26 ± 1
Met‐Lys‐ACC	4	1.00 ± 0.04	34 ± 2	29 ± 1
Met‐Bpa‐ACC	3	0.48 ± 0.04	14 ± 3	34 ± 4
Met‐hArg‐ACC	4	0.92 ± 0.02	27 ± 2	35 ± 1
Met‐Arg‐ACC	4	1.26 ± 0.06	31 ± 3	41 ± 2
Met‐Glu(Bzl)‐ACC	4	0.56 ± 0.04	13 ± 2	46 ± 6
Met‐Arg(NO_2_)‐ACC	4	1.90 ± 0.08	23 ± 2	84 ± 4
Met‐nLeu(o‐Bzl)‐ACC	10	1.66 ± 0.02	8.8 ± 0.4	186 ± 6
hPro‐hPhe‐ACC^a^	3	0.007 ± 0.001	26 ± 6	0.30 ± 0.04
Trp‐hPhe‐ACC	3	0.017 ± 0.002	7 ± 1	2.5 ± 0.3
Phe‐hPhe‐ACC	3	0.032 ± 0.008	20 ± 8	1.6 ± 0.2
Igl‐hPhe‐ACC	3	0.044 ± 0.004	4.7 ± 0.9	9.4 ± 1
hAla‐hPhe‐ACC	3	0.0014 ± 0.002	14.7 ± 0.8	9.8 ± 1
Ile‐hPhe‐ACC	4	0.12 ± 0.01	12 ± 3	10 ± 1
Phg‐hPhe‐ACC	4	0.110 ± 0.004	10 ± 1	11 ± 1
Phe(Me)‐hPhe‐ACC	3	0.072 ± 0.004	6 ± 1	12 ± 1
Tyr(NO_2_)‐hPhe‐ACC	3	0.134 ± 0.002	9.9 ± 0.4	13.6 ± 0.4
Gln‐hPhe‐ACC	3	0.052 ± 0.001	19 ± 10	2.7 ± 0.9
Glu‐hPhe‐ACC	3	0.024 ± 0.002	1.7 ± 0.5	13 ± 3
Val‐hPhe‐ACC	3	0.24 ± 0.04	14 ± 4	18 ± 2
nLeu‐hPhe‐ACC	3	0.28 ± 0.02	5.9 ± 1.3	46 ± 6
nVal‐hPhe‐ACC	4	0.164 ± 0.005	1.30 ± 0.08	128 ± 6
Leu‐hPhe‐ACC	3	0.100 ± 0.002	0.50 ± 0.03	210 ± 10
Phg‐nLeu(o‐Bzl)‐ACC	8	0.80 ± 0.02	7.8 ± 0.8	102 ± 6
Leu‐nLeu(o‐Bzl)‐ACC	5	0.308 ± 0.006	0.5 ± 0.1	640 ± 100
nVal‐nLeu(o‐Bzl)‐ACC	8	1.28 ± 0.02	1.4 ± 0.1	920 ± 60
Leu‐Bpa‐ACC	4	0.170 ± 0.004	0.61 ± 0.06	280 ± 20
Phe‐Arg‐ACC	4	0.018 ± 0.006	20 ± 10	0.9 ± 0.2
Pro‐Arg‐AMC^b^	3	0.102 ± 0.004	88 ± 5	1.16 ± 0.04
Val‐Arg‐ACC^c^	3	1.08 ± 0.06	63 ± 5	17.0 ± 0.4
Leu‐Arg‐ACC	8	0.330 ± 0.006	1.8 ± 0.2	185 ± 15
nVal‐Arg‐ACC	8	0.346 ± 0.004	0.72 ± 0.05	480 ± 30
Phe‐Arg‐βNA	4	0.96 ± 0.04	51 ± 5	19 ± 1

*N* is the number of replicates. Standard errors are shown for each parameter.

a For DPAP1, *k*_cat _= 0.22 ± 0.004 s^−1^, *K*_m _= 0.67 ± 0.14 μm and *k*_cat_/*K*_m_ = 320 000 ± 50 000 m ^−1^·s^−1^; for CatC, *k*_cat_ = 10.8 ± 0.2 s^−1^, *K*_m_ = 91 ± 5 μm and *k*_cat_/*K*_m_ = 116 000 ± 11 000 m ^−1^·s^−1^ 35.

b For DPAP1, *k*_cat_ = 6.2 ± 0.4 s^−1^, *K*_m_ = 84 ± 9 μm and *k*_cat_/*K*_m_ = 74 000 ± 2000 m ^−1^·s^−1^; for CatC, *k*_cat_ = 490 ± 10 s^−1^, *K*_m_ = 130 ± 10 μm and *k*_cat_/*K*_m_ = 3 600 000 ± 300 000 m ^−1^·s^−1^ 41.

c For DPAP1, *k*_cat_ = 3.5 ± 0.1 s^−1^, *K*_m_ = 21 ± 2 μm and *k*_cat_/*K*_m_ = 170 000 ± 10 000 m ^−1^·s^−1^; for CatC, *k*_cat_ = 180 ± 10 s^−1^, *K*_m_= 51 ± 8 μm and *k*_cat_/*K*_m_ =3600 000 ± 300 000 m ^−1^·s^−1^ 41.

P1 residues have a significant influence in *k*_cat_, with nLeu(o‐Bzl) and positively charged residues having the highest values. A positive charge on the δ position (Arg(NO_2_) and Arg) is favoured over the ε position (Lys and hArg). Elongated aliphatic and hydrophobic residues in P1 decrease *K*_m_, especially when aromatic groups are distant from the peptide backbone. This is evident by the decreasing *K*_m_ values between nVal, Met, hPhe, Bpa, Glu(Bzl) and nLeu(o‐Bzl). This tendency was also observed for CatC and DPAP1 and might suggest the presence of a distal binding pocket (Fig. 1B), potentially an exosite, since P1 residues are usually solvent exposed in clan CA proteases.

P2 residues have a bigger influence on *K*_m_ than P1, with Leu and nVal being optimal P2 residues for DPAP3. Beta‐branched residues are not optimal for DPAP3 as can be observed by an increase in *K*_m_ between nVal and Val or nLeu and Ile. However, the γ‐branched AA Leu has the lowest *K*_m_ value. Substrates with aliphatic P2 side chains that extend to the δ position (Met and nLeu) result in higher *K*_m_ values than slightly shorter ones (Leu and nVal) but also higher *k*_cat_ values. Overall, combining optimal P1 (nLeu(o‐Bzl) and Arg) and P2 (nVal and Leu) residues results in improved *k*_cat_/*K*_m_ values (Table 1).

Interestingly, although substrates with Phg and indanyl‐glycine (Igl) in P2, or Bpa in P1, are not the preferred AAs at these positions, these non‐natural residues are structurally very different from natural AAs and are turned over quite efficiently by DPAP3 when combined with optimal P1 or P2 residues respectively, that is, Leu‐Bpa‐ACC or Phg‐nLeu(o‐Bzl)‐ACC. Finally, the optimal substrate for DPAP1, hPro‐hPhe‐ACC 35 is very poorly turned over by DPAP3 (> 200‐fold difference in *k*_cat_/*K*_m_). We think that substrates containing these non‐natural AAs could be used as specific tools to measure DPAP1 or DPAP3 activity in biological samples, that is, parasite lysates or live parasites, an application we are currently investigating.

**Figure 2:**
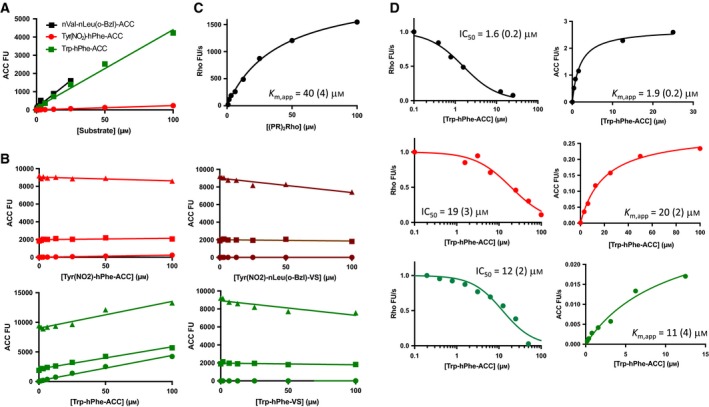
Substrate quenching and substrate competition studies. (A) Background fluorescence signal of selected substrates measured in assay buffer. Note that Tyr(NO_2_)‐hPhe‐ACC shows a very low level of background fluorescence compared to Trp‐hPhe‐ACC or nVal‐nLeu(o‐Bzl)‐ACC (Fig. 3A) likely indicating intramolecular quenching between ACC and Tyr(NO_2_). (B) ACC fluorescence signal measured at increasing concentrations of the indicated substrates and inhibitors in the presence at 0 (circles), 1 (squares) or 5 (triangles) μm of free ACC. (C) (PR)_2_Rho turnover by DPAP3. (D) Turnover rates of (PR)_2_Rho (left graphs) and ACC substrates (right graphs) measured at 40 μm of (PR)_2_Rho and increasing concentrations of ACC substrates. *K*_m,app_ and IC_50_ values are indicated in each graph. Numbers in parentheses represent the standard error of the fit (*N* = 1).

Finally, our studies show that substrates with aromatic P2 residues are poorly cleaved by DPAP3 compared to optimal substrates, that is, 100 to 1000‐fold lower *k*_cat_/*K*_m_. This is surprising since vinyl sulfone inhibitors containing aromatic P2 residues such as Tyr(NO_2_) or Trp are potent and selective DPAP3 inhibitors 21, 26. Because these two AA side chains have fluorogenic properties, we investigated whether the low turnover rate measured for Tyr(NO_2_)‐hPhe‐ACC and Trp‐hPhe‐ACC might be due to quenching effects. The emission of free ACC (0, 1, or 5 μm) in assay buffer was measured in the presence of 0‐100 μm of these substrates (Fig. 2A,B). No significant decrease in the ACC emission signal was observed even when substrates were present in 100‐fold excess, thus indicating that the low turnover rates measured for these substrates are not due to quenching effects.

**Figure 3:**
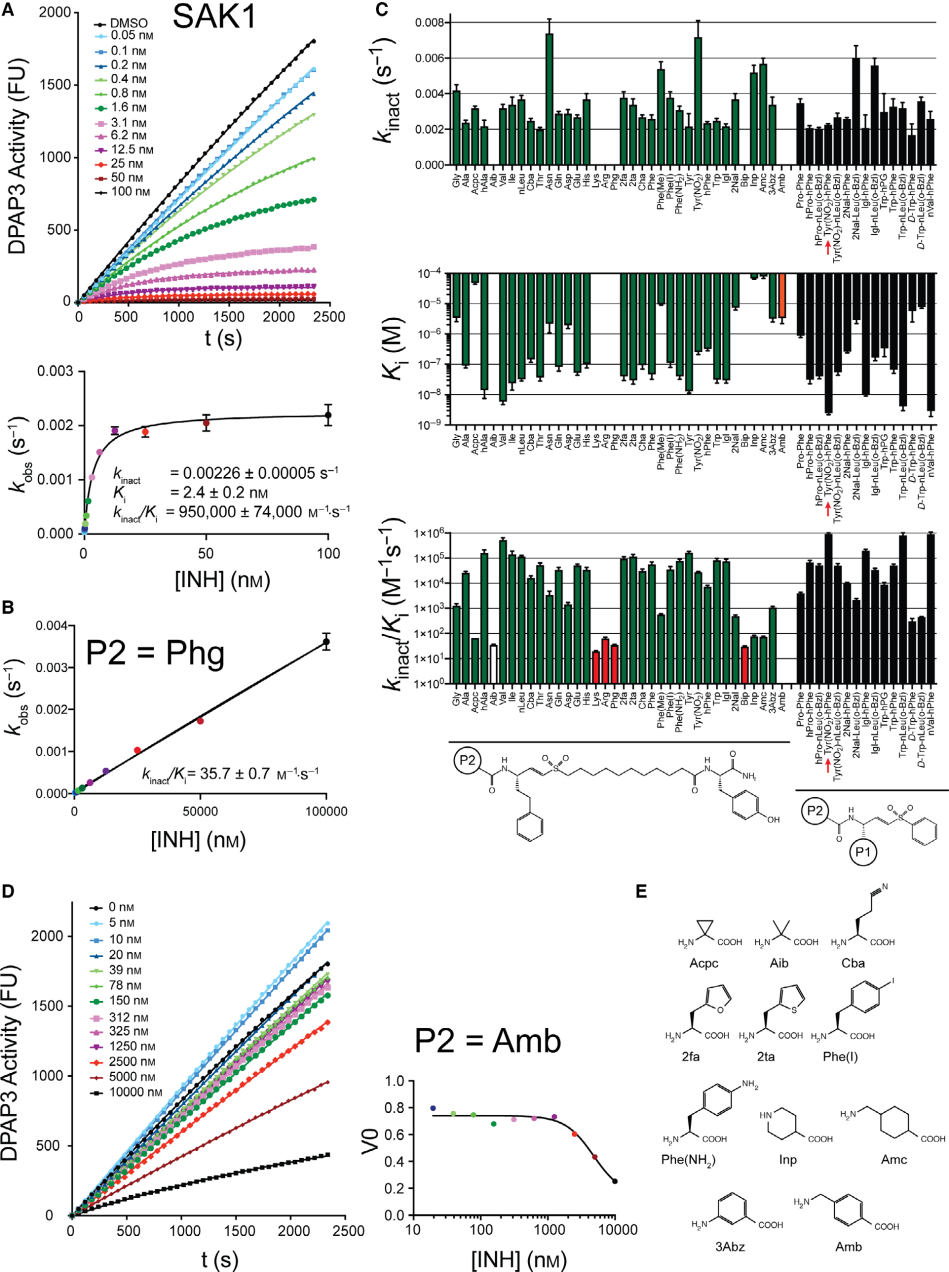
DPAP3 inhibitor specificity. (A) Representative data showing time‐dependent inhibition of DPAP3 by SAK1 (Tyr(NO_2_)‐hPhe‐VS). Each progress curve (FU vs. time) was fitted to Eqn. 5 to obtain *k*_obs_ values (top graph). These were then fitted to Eqs. 6 and 7 to obtain *k*_inact_, *K*_i_ and *k*_inact_/*K*_i_. Progress curves and corresponding *k*_obs_ values at each inhibitor concentration are shown in different colours. (B) Example of an inhibitor where no inhibitor saturation was observed (white bars in C). In this case, *k*_obs_ values were fitted to a linear model to obtain *k*_inact_/*K*_i_. (C) Inhibition constants determined for DPAP3. The general structure of the inhibitors is shown below the graphs. Red bars correspond to inhibitors for which only *k*_inact_/*K*_i_ values could be determined. The orange bar corresponds to the only inhibitor that showed a reversible mechanism of inhibition, that is, only a *K*_i_ value could be determined. The inhibitor corresponding to SAK1 is indicated with a red arrow. (D) No time‐dependence inhibition of DPAP3 was observed with an inhibitor with a P2 Amb. Initial turnover rates (*V*_0_) were fitted to a reversible binding model to obtain *K*_i_. Progress curves and corresponding *V*_0_ values at each inhibitor concentration are shown in different colours. (E) Structure of non‐natural AAs present in the inhibitor library but not in the substrate library. Error bars represent the standard error of the global fit for each parameter obtained by fitting *k*_obs_ values vs. inhibitor concentration (1 or 2 technical replicates per compound) to Eqs. 6 or 7.

As an alternative method to confirm that Tyr(NO_2_)‐hPhe‐ACC and Trp‐hPhe‐ACC bind relatively poorly to DPAP3, we performed substrate competition assays using the (PR)_2_Rho substrate (λ_ex _= 492 nm, λ_em _= 523 nm), which emits at much higher wavelengths than ACC (λ_ex _= 355 nm, λ_ex _= 460 nm), thus allowing us to simultaneously measure the turnover of (PR)_2_Rho and ACC substrates without quenching interference. (PR)_2_Rho was initially designed as a DPAP1‐specific substrate to directly measure the activity of this protease in crude parasite extracts 42, but it is also cleaved by DPAP3 with a *K*_m,app_ of 40 μm (Fig. 3C). This substrate is cleaved twice by DPAPs, releasing two Pro‐Arg dipeptides and the rhodamine 110 fluorophore. In this assay, we simultaneously measured inhibition of (PR)_2_Rho turnover by ACC substrates (IC_50_ values) as well as the apparent *K*_m_ values of these ACC substrates in the presence of 40 μm (PR)_2_Rho. As shown in Fig. 2D, the IC_50_ and *K*_m,app_ values obtained are within experimental error and, as expected, slightly higher than the *K*_m_ values reported in Table 1 due to the substrate competition effect.

Overall, the lack of quenching effect between Trp or Tyr(NO_2_) and ACC, and the good agreement between IC_50_ and *K*_m,app_ measured under substrate competition conditions indicates that the Michaelis–Menten parameters reported in Table 1 are accurate and that substrates containing aromatic P2 AAs are indeed relatively poor DPAP3 substrates compared to those containing optimal aliphatic P2 residues. These results raise the question of why vinyl sulfone inhibitors with aromatic P2 residues are among the most potent DPAP3 inhibitors identified so far. To better understand this discrepancy, we measured the kinetics of inactivation of DPAP3 by the previously published vinyl sulfone inhibitor library 25, 26.

### DPAP3 inhibitor specificity

Time‐ and concentration‐dependent inactivation of DPAP3 by the P2 library of vinyl sulfone inhibitors (P1 fixed to hPhe) was measured using a continuous assay at 2.2 μm of Met‐nLeu(o‐Bzl)‐ACC (0.25 x *K*_m_). For most compounds, the mechanism of inhibition was consistent with a two‐step irreversible inhibition model (Eqn. (1), Fig. 3A).

(1) $$E+I\overset{K_{i}}{⇄}E:I\overset{k_{\mathrm{inact}}}{\to }E-I$$

*K*_i_ is the inhibition equilibrium constant, and *k*_inact_ the rate of covalent modification of the catalytic cysteine.

For a few inhibitors only *k*_inact_/*K*_i_ values could be obtained, that is, no saturation was achieved in the *k*_obs_ *vs*. [I] graph (Fig. 3B). The inhibition constants are reported in Fig. 3C and Table 2 (See also Fig. S2 for representative fits). Only one inhibitor, containing an amino‐1‐methyl‐benzyl (Amb) group in P2, was not able to inhibit DPAP3 in a time‐dependent manner under our assay conditions (Fig. 3D). This is probably due to the fact that this extended and rigid P2 AA (Fig. 3E) might prevent proper positioning of the vinyl sulfone group into the active site of DPAP3 to allow covalent modification of the catalytic cysteine. For this compound, a *K*_i_ value for reversible inhibition was measured.

**Table 2:** DPAP3, DPAP1 and CatC inhibition constants for the vinyl sulfone library

P2	DPAP3			DPAP1			CatC		
	*k*_inact_ ·10^3^ (s^−1^)	*K*_i_ (nm)	*k*_inact_/*K*_i_·10^‐3^ (m ^−1^·s^−1^)	*k*_inact_ ·10^3^ (s^−1^)	*K*_i_ (nm)	*k*_inact_/*K*_i_·10^‐3^ (m ^−1^·s^−1^)	*k*_inact_ ·10^3^ (s^−1^)	*K*_i_ (nm)	*k*_inact_/*K*_i_·10^−3^ (m ^−1^·s^−1^)
Gly	4.2 ± 0.3	3300 ± 750	1.30 ± 0.25	3.0 ± 0.2	3300 ± 750	0.9 ± 0.1	3.0 ± 0.2	70 ± 9	43 ± 4
Ala	2.4 ± 0.1	88 ± 13	27 ± 3	3.03 ± 0.09	90 ± 7	34 ± 2	3.00 ± 0.06	9.7 ± 0.4	310 ± 8
Acpc	3.2 ± 0.1	47 000 ± 3000	0.0677 ± 0.0001	6.2 ± 0.3	49 000 ± 4000	0.127 ± 0.006	1.03 ± 0.06	3200 ± 700	0.33 ± 0.06
hAla	2.2 ± 0.3	13.5 ± 6	166 ± 50	5.2 ± 0.7	19 ± 4	267 ± 26	2.9 ± 0.2	5.9 ± 0.9	500 ± 50
Aib	N.S.	N.S.	0.0363 ± 0.0006	8.9 ± 0.5	18 000 ± 2000	0.49 ± 0.03	1.20 ± 0.07	2100 ± 300	0.57 ± 0.06
Val	3.2 ± 0.2	5.7 ± 1.1	554 ± 84	3.6 ± 0.2	2.6 ± 0.4	1380 ± 140	2.9 ± 0.1	14 ± 2	200 ± 15
Ile	3.4 ± 0.4	23 ± 9	148 ± 45	3.2 ± 0.1	92 ± 8	34 ± 3	3.7 ± 0.8	1300 ± 400	2.9 ± 0.3
nLeu	3.7 ± 0.2	31 ± 3	120 ± 7.5	4.4 ± 0.3	14 ± 2	305 ± 24	2.40 ± 0.05	7.0 ± 0.5	340 ± 20
Cba	2.5 ± 0.1	140 ± 25	17 ± 2.5	5.4 ± 0.4	13 000 ± 2000	0.41 ± 0.03	1.29 ± 0.03	2400 ± 140	0.53 ± 0.02
Thr	2.0 ± 0.1	36 ± 8	55 ± 10	4.4 ± 0.2	112 ± 10	39 ± 2	2.38 ± 0.03	65 ± 3	36 ± 1
Asn	7.4 ± 0.8	2100 ± 1000	3.5 ± 1.4	9.9 ± 0.5	10 500 ± 750	0.95 ± 0.02	2.40 ± 0.07	260 ± 30	9.2 ± 0.7
Gln	2.9 ± 0.2	80 ± 20	35 ± 8	4.7 ± 0.2	50 ± 3	93 ± 3	2.3 ± 0.1	15 ± 3	160 ± 25
Asp	2.9 ± 0.2	1900 ± 400	1.5 ± 0.2	5.4 ± 0.2	2100 ± 200	2.6 ± 0.10	3.0 ± 0.1	2200 ± 200	1.4 ± 0.2
Glu	2.7 ± 0.1	51 ± 8	53 ± 6	5.0 ± 0.4	650 ± 100	7.6 ± 0.75	2.6 ± 0.1	140 ± 20	18 ± 2
His	3.7 ± 0.3	100 ± 25	35.5 ± 7.5	4.3 ± 0.3	150 ± 20	29 ± 3	2.1 ± 0.1	0.6 ± 0.1	3350 ± 500
Lys	N.S.	N.S.	0.020 ± 0.001	N.S.	N.S.	0.081 ± 0.004	1.49 ± 0.05	3600 ± 200	0.42 ± 0.02
Arg	N.S.	N.S.	0.065 ± 0.006	N.S.	N.S.	0.084 ± 0.002	4.0 ± 0.5	11 000 ± 2000	0.38 ± 0.02
Phg	N.S.	N.S.	0.0357 ± 0.0007	8.3 ± 0.5	7000 ± 650	1.19 ± 0.05	2.5 ± 0.2	86 ± 20	29 ± 5
2fa	3.8 ± 0.3	39 ± 10	97 ± 18	5.2 ± 0.4	11 ± 3	470 ± 90	3.7 ± 0.2	2.1 ± 0.2	1720 ± 90
2ta	3.4 ± 0.3	29 ± 7	118 ± 23	3.8 ± 0.3	82 ± 20	47 ± 7	3.3 ± 0.3	6.5 ± 1	510 ± 55
Cha	2.7 ± 0.1	90 ± 20	31.6 ± 5.5	N.S.	N.S.	0.020 ± 0.001	2.3 ± 0.1	300 ± 40	7.7 ± 0.8
Phe	2.6 ± 0.2	45 ± 13	60 ± 15	N.S.	N.S.	0.40 ± 0.01	2.18 ± 0.08	1.4 ± 0.2	1600 ± 200
Phe(Me)	5.4 ± 0.4	9200 ± 300	0.580 ± 0.025	N.S.	N.S.	0.314 ± 0.005	2.8 ± 0.2	1300 ± 200	2.2 ± 0.3
Phe(I)	3.8 ± 0.3	110 ± 30	36 ± 10	7.0 ± 0.3	47 000 ± 3500	0.151 ± 0.006	1.02 ± 0.06	185 ± 40	5.5 ± 0.9
Phe(NH2)	3.1 ± 0.2	39 ± 7	80 ± 12	5.0 ± 0.4	22 000 ± 2500	0.23 ± 0.01	1.01 ± 0.02	5.3 ± 0.3	191 ± 9
Tyr	2.18 ± 0.07	12.7 ± 1.5	172 ± 16	N.S.	N.S.	0.310 ± 0.005	2.10 ± 0.06	11.5 ± 0.9	182 ± 10
Tyr(NO_2_)	7.2 ± 0.9	250 ± 40	28.8 ± 0.8	N.S.	N.S.	0.023 ± 0.002	N.S.	N.S.	0.147 ± 0.008
hPhe	2.36 ± 0.08	310 ± 30	7.6 ± 0.7	N.S.	N.S.	0.029 ± 0.002	N.S.	N.S.	0.103 ± 0.005
Trp	2.5 ± 0.1	30 ± 6	84 ± 14	N.S.	N.S.	0.074 ± 0.002	1.98 ± 0.03	61 ± 4	32 ± 2
Igl	2.2 ± 0.1	29 ± 5	78 ± 12	N.S.	N.S.	0.313 ± 0.007	2.3 ± 0.1	240 ± 40	10 ± 1
2Nal	3.7 ± 0.3	7100 ± 1000	0.510 ± 0.04	5.3 ± 1.3	15 200 ± 6500	0.35 ± 0.07	2.6 ± 0.2	1800 ± 400	1.5 ± 0.2
Bip	N.S.	N.S.	0.0314 ± 0.0004	N.S.	N.S.	0.0178 ± 0.0004	2.53 ± 0.09	6200 ± 800	0.41 ± 0.04
Inp	5.2 ± 0.4	65 000 ± 800	0.079 ± 0.004	N.S.	N.S.	0.03 ± 0.001	1.05 ± 0.06	5600 ± 900	0.19 ± 0.02
Amc	5.7 ± 0.3	75 000 ± 7000	0.076 ± 0.003	5.8 ± 0.3	27 000 ± 2500	0.213 ± 0.009	0.97 ± 0.04	1000 ± 200	0.94 ± 0.17
3Abz	3.4 ± 0.4	3100 ± 600	1.1 ± 0.1	4.8 ± 0.3	3000 ± 500	1.6 ± 0.2	1.08 ± 0.07	55 ± 12	20 ± 4
Amb	N/A	3200 ± 1000	N/A	N.S.	N.S.	0.140 ± 0.007	1.6 ± 0.1	2100 ± 300	0.74 ± 0.06

N.S. No saturation, only *k*_inact/_ *K*_i_ could be obtained. N/A, no time‐dependent inactivation was observed, data consistent with reversible inhibition. Standard error of the global fit for each parameter and inhibitor obtained by fitting *k*_obs_ values (1 or 2 technical replicates for DPAP3, single replicates for DPAP1 and CatC) to Eqs. 6 or 7 are shown.

**Figure 4:**
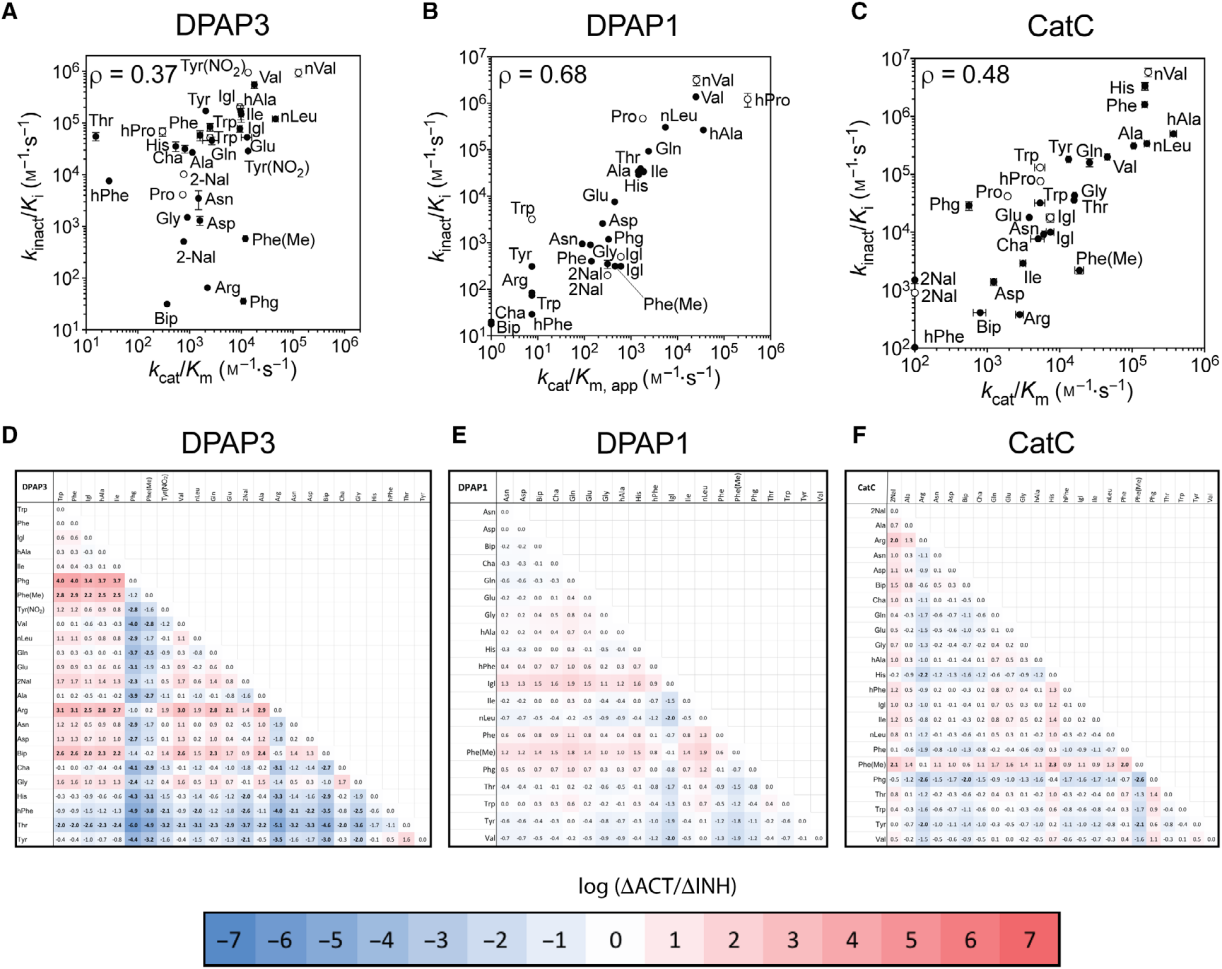
Comparison of catalytic efficiency and second‐order inhibition constants. (A–C) Comparison of *k*_inact_/*K*_i_ and *k*_cat_/*K*_m_ for DPAP3 (A), DPAP1 (B) and CatC (C) between vinyl sulfone inhibitors and P2 substrate library. For DPAP1, apparent *k*_cat_/*K*_m_ (*k*_cat_/*K*_m,app_) were calculated based on previously reported turnover rates at 1 μm 35. *k*_cat_/*K*_m,app_ were calculated similarly for DPAP3 and CatC for P2 substrates whose activity was too low to obtain accurate Michaelis–Menten parameters (i.e., substrates not present in Tables 1 and S2). Filled circles correspond to compounds belonging to the vinyl sulfone library (compounds in Table 2), and empty circles to inhibitors having a phenyl group in P1′ (Table 4). The P2 residue is labelled next to each data point. Pearson correlation coefficients (ρ) are shown for each protease and were calculated using the default function in Prism. Error bar represents the standard error of the fit for each parameter. (D–F) Comparison of changes in substrate turnover relative to inhibitor potency for any pair of P2 residues for the P2 substrate library and the VS inhibitor library (both with P1 hPhe). The log value of ΔACT/ΔINH (Eqn. 2) calculated for DPAP3 (D), DPAP1 (E) and CatC (F) are shown as a heat maps with values above and below zero in red and blue respectively. Each pairwise value showing more than a 100‐fold discrepancy between activity and inhibition (ΔACT/ΔINH > 100 or < 0.01) is highlighted in bold.

Overall, changes in P2 do not have a big influence in *k*_inact_ with the exceptions of Asn, Phe(Me) and Tyr(NO_2_), which significantly increase *k*_inact_. Intriguingly, substrates containing the latter two P2 residues were the only substrates with a P2 aromatic residue that could be cleaved by DPAP3 with *k*_cat_/*K*_m_ > 2000 m ^−1^·s^−1^ (Table 1). In terms of *K*_i_, we observed some SAR similarities between substrates and inhibitors: DPAP3 does not bind inhibitors with an N‐terminal basic residue (Arg or Lys), but it is strongly inhibited (*K*_i_ < 35 nm) by aliphatic residues (Leu, nLeu, hAla, or Val). However, we measured potent inhibition of DPAP3 with aromatic residues in P2 such as Phe, Tyr or Trp (*k*_inact_/*K*_i_ ≥ 60 000 m ^−1^·s^−1^). Substrates with these P2 residues show relatively poor substrate turnover (*k*_cat_/*K*_m_ ≤ 2000 m ^−1^·s^−1^). We also observed other discrepancies between substrates and inhibitors. For example, Thr in P2 results in a poor substrate (Fig. 1B) but a relatively potent inhibitor (*k*_inact_/*K*_i_ = 55 000 m ^−1^·s^−1^). Inversely, DPAP3 cleaves Phg‐hPhe‐ACC and Phe(Me)‐hPhe‐ACC with a *k*_cat_/*K*_m_ of 11 000 and 12 000 m ^−1^·s^−1^, respectively, but the *k*_inact_/*K*_i_ for the respective inhibitors are only 36 and 580 m ^−1^·s^−1^. As shown in Fig. 4A, there is not a clear correlation between *k*_cat_/*K*_m_ and *k*_inact_/*K*_i_ for DPAP3 as a function of the P2 residue, however, P2 residues that make optimal substrates also make good inhibitors (Val, nVal, nLeu).

### Correlation between substrate turnover and inhibition for DPAP1 and CatC

To determine whether the lack of correlation between *k*_cat_/*K*_m_ and *k*_inact_/*K*_i_ observed for DPAP3 is a common feature in DPAPs, we calculated apparent *k*_cat_/*K*_m_ values for DPAP1 for the P2 substrate library based on the activity measurements previously reported at 1 μm and the *k*_cat_/*K*_m_ for hPro‐hPhe‐ACC 35. We also measured Michaelis–Menten parameters for CatC for the P2 substrate library (Table 3 and Fig. S3), and *k*_inact_ and *K*_i_ values for DPAP1 and CatC for the P2 vinyl sulfone library (Table 2 and Fig. S2). As shown in Fig. 4B,C, we observed a good correlation between substrate turnover and *k*_inact_/*K*_i_ for DPAP1 and CatC. However, a few discrepancies were observed for CatC (Fig. 4C). For example, the *k*_cat_/*K*_m_ for Phe(Me)‐hPhe‐ACC is 30‐fold higher than for Phg‐hPhe‐ACC while the *k*_inact_/*K*_i_ for Phe(Me)‐hPhe‐VS is 13‐fold lower than for Phg‐hPhe‐VS, thus resulting in a 400‐fold discrepancy in the changes in *k*_cat_/*K*_m_ and *k*_inact_/*K*_i_.

**Table 3:** Steady‐state Michaelis–Menten parameters for CatC

Substrate	N	*k*_cat_ (s^−1^)	*K*_m_ (μm)	*k*_cat_/*K*_m_ ·10^‐3^ (m ^−1^·s^−1^)
Ala‐hPhe‐ACC	3	2.13 ± 0.07	20 ± 2	105 ± 7
Arg‐hPhe‐ACC	3	0.26 ± 0.05	90 ± 25	2.8 ± 0.4
Asp‐hPhe‐ACC	3	N.S.	N.S.	1.2 ± 0.1
Glu‐hPhe‐ACC	3	N.S.	N.S.	3.8 ± 0.1
Asn‐hPhe‐ACC	3	N.S.	N.S.	6.0 ± 0.3
Gln‐hPhe‐ACC	3	1.52 ± 0.08	59 ± 5	26 ± 1
Gly‐hPhe‐ACC	3	1.6 ± 0.1	100 ± 10	16.1 ± 0.8
His‐hPhe‐ACC	3	1.23 ± 0.05	8 ± 1	150 ± 14
Ile‐hPhe‐ACC	3	0.21 ± 0.03	68 ± 15	3.1 ± 0.3
Phe‐hPhe‐ACC	3	0.68 ± 0.03	4.5 ± 0.5	150 ± 15
Pro‐hPhe‐ACC	3	N.S.	N.S.	1.9 ± 0.1
Thr‐hPhe‐ACC	3	0.68 ± 0.03	43 ± 3	15.8 ± 0.7
Trp‐hPhe‐ACC	3	0.30 ± 0.05	56 ± 20	534 ± 0.9
Tyr‐hPhe‐ACC	3	0.37 ± 0.02	28 ± 3	13.3 ± 0.8
Val‐hPhe‐ACC	3	1.45 ± 0.08	32 ± 4	46 ± 4
hPhe‐hPhe‐ACC	3	N.A.	N.A.	N.A.
Cha‐hPhe‐ACC	3	0.022 ± 0.003	4.5 ± 1.5	5 ± 1
nLeu‐hPhe‐ACC	3	1.92 ± 0.08	12 ± 1	160 ± 15
Phg‐hPhe‐ACC	3	N.S.	N.S.	0.58 ± 0.05
Bip‐hPhe‐ACC	3	N.S.	N.S.	0.8 ± 0.2
Igl‐hPhe‐ACC	3	0.11 ± 0.02	15 ± 4	7.4 ± 0.9
Phe(Me)‐hPhe‐ACC	3	0.084 ± 0.008	4.5 ± 1	19 ± 3
2Nal‐hPhe‐ACC	3	N.A.	N.A.	N.A.
hAla‐hPhe‐ACC	3	2.05 ± 0.08	5.5 ± 0.8	372 ± 40
nVal‐hPhe‐ACC	3	2.06 ± 0.04	12.3 ± 0.6	167 ± 6
hPro‐hPhe‐ACC	3	0.8 ± 0.1	150 ± 30	5.4 ± 0.3

N is the number of replicates. N.A. No activity measured up to 100 μm substrate. Standard errors are shown for each parameter.

A systematic way to visualize discrepancies between substrate turnover and inhibition is to compare the fold difference in *k*_cat_/*K*_m_ with that in *k*_inact_/*K*_i_ for any two P2 residues (Eqn. 2).

(2) $$\frac{\Delta \text{ACT}}{\Delta \text{INH}}=\frac{{\left(k_{\mathrm{cat}}/K_{m}\right)}_{\mathrm{AA}1}/{\left(k_{\mathrm{cat}}/K_{m}\right)}_{\mathrm{AA}2}}{{\left(k_{\mathrm{inact}}/K_{m}\right)}_{AA1}/{\left(k_{\mathrm{inact}}/K_{m}\right)}_{\mathrm{AA}2}}$$

We performed these pairwise calculations for each of the DPAPs studied here and have presented the results as heat maps in Fig. 4D–F. We observed significant and numerous discrepancies between substrates and inhibitors for DPAP3 (30% of pairwise ΔACT/ΔINH > 100 or < 0.01), almost no discrepancies for DPAP1 (only 1% ΔACT/ΔINH > 100 or < 0.01), and only a few for CatC (4% of ΔACT/ΔINH > 100 or < 0.01). Overall, this study indicates that the level of correlation between *k*_cat_/*K*_m_ and *k*_inact_/*K*_i_ is protease‐dependent.

### Development of DPAP1 and DPAP3‐selective inhibitors

We next synthesized several inhibitors to determine whether the optimal nLeu(o‐Bzl) P1 residue identified from the substrate screen could be used to increase the potency and specificity of inhibitors towards DPAP1 or DPAP3. We selected P2 AAs that were predicted to provide specificity towards DPAP1 (Pro and hPro) or DPAP3 (aromatic residues: Tyr(NO_2_), Trp, Igl and 2Nal) based on our substrate and inhibitor screening results. We also included in our analysis the previously synthesized compound JCP410 (nVal‐hPhe‐VS) 26, since nVal is one of the best P2 residues identified from the substrate screen. We determined the inhibition constants of these compounds for DPAP1, DPAP3 and CatC (Table 4 and Figs 3 and S2). The major structural difference between these compounds and the inhibitor library is that they have a phenyl group in P’ instead of a long aliphatic linker (Fig. 3A). This change usually increases the potency of compounds except in the context of a P2 Trp for DPAP3, or P2 Tyr(NO_2_) or 2Nal for CatC (Tables 2 and 3). These exceptions indicate some level of interdependence between the prime and nonprime sites of DPAPs.

**Table 4:** Inhibition constants for optimal vinyl sulfone inhibitors

Inhibitor Name	DPAP3			DPAP1			CatC			Specificity ratio^a^		
	*k*_inact_ ·10^3^ (s^−1^)	*K*_i_ (nm)	*k*_inact_/*K*_i_ ·10^−3^ (m ^−1^·s^−1^)	*k*_inact_ ·10^3^ (s^−1^)	*K*_i_ (nm)	*k*_inact_/*K*_i_ ·10^−3^ (m ^−1^·s^−1^)	*k*_inact_ ·10^3^ (s^−1^)	*K*_i_ (nm)	*k*_inact_/*K*_i_ ·10^−3^ (m ^−1^·s^−1^)	DPAP1/DPAP3	DPAP1/CatC	DPAP3/CatC
Pro‐hPhe‐VS	3.5 ± 0.2	860 ± 100	4.1 ± 0.3	2.1 ± 0.2	4.6 ± 0.7	470 ± 35	1.45 ± 0.07	34 ± 5	42 ± 5	**115**	11	0.10
hPro‐hPhe‐VS	2.1 ± 0.1	30 ± 7	69 ± 13	4.8 ± 0.6	3.9 ± 1.5	1230 ± 400	1.67 ± 0.08	22 ± 3	76 ± 0.8	18	16	0.91
hPro‐nLeu(o‐Bzl)‐VS	2.0 ± 0.1	39 ± 6	53 ± 6.5	2.9 ± 0.3	0.8 ± 0.2	3560 ± 150	1.67 ± 0.03	11.2 ± 0.6	150 ± 6	68	24	0.35
Tyr(NO_2_)‐hPhe‐VS	2.26 ± 0.05	2.4 ± 0.2	950 ± 74	3.8 ± 1	25 000 ± 1400	0.15 ± 0.05	3.2 ± 0.6	58 000 ± 20 000	0.054 ± 0.008	**0.00016**	2.8	**17 600**
Tyr(NO_2_)‐nLeu(o‐Bzl)‐VS	2.7 ± 0.2	53 ± 10	51 ± 10	2.6 ± 0.3	2400 ± 900	1.1 ± 0.3	2.9 ± 0.2	7700 ± 1400	0.38 ± 0.05	0.032	2.9	89
2Nal‐hPhe‐VS	2.59 ± 0.07	254 ± 16	10.2 ± 0.5	6.7 ± 0.2	33 000 ± 2000	0.2 ± 0.005	3.7 ± 0.2	4000 ± 700	0.9 ± 0.1	0.02	0.22	11
2Nal‐nLeu(o‐Bzl)‐VS	6.0 ± 0.7	2800 ± 600	2.2 ± 0.3	5.0 ± 1.7	27 000 ± 16 000	0.19 ± 0.05	2.4 ± 0.6	1740 ± 140	1.37 ± 0.09	0.14	0.14	1.0
Igl‐hPhe‐VS	2.1 ± 0.7	10 ± 1	205 ± 22	2.7 ± 0.1	5400 ± 500	0.50 ± 0.03	5.8 ± 0.3	320 ± 70	18 ± 3	**0.0024**	0.028	11.4
Igl‐nLeu(o‐Bzl)‐VS	5.6 ± 0.4	160 ± 30	35 ± 4.5	4.1 ± 0.2	4800 ± 500	0.86 ± 0.05	1.48 ± 0.06	11 ± 2	135 ± 14	0.037	**0.0064**	0.17
*L‐*Trp‐hPG‐VS	3 ± 1	330 ± 150	9.1 ± 1.5	2.1 ± 0.2	2800 ± 450	0.74 ± 0.07	1.73 ± 0.03	28 ± 2	61 ± 4	0.12	0.012	0.10
*L‐*Trp‐hPhe‐VS	3.3 ± 0.4	65 ± 15	52 ± 7	1.7 ± 0.1	540 ± 80	3.20 ± 0.3	2.2 ± 0.09	16 ± 3	131 ± 15	0.094	0.024	0.26
*L‐*Trp‐nLeu(o‐Bzl)‐VS	3.2 ± 0.3	4 ± 1	826 ± 193	2.2 ± 0.1	380 ± 60	5.8 ± 0.6	1.74 ± 0.03	4.9 ± 0.3	355 ± 18	**0.0070**	0.016	2.3
*D‐*Trp‐hPhe‐VS	1.7 ± 0.6	5500 ± 3000	0.32 ± 0.08	2.7 ± 0.2	105 000 ± 10 000	0.026 ± 0.001	2.6 ± 0.1	4200 ± 600	0.62 ± 0.07	0.12	0.042	0.34
*D‐*Trp‐nLeu(o‐Bzl)‐VS	3.6 ± 0.2	7600 ± 600	0.47 ± 0.01	5.5 ± 0.7	127 000 ± 20 000	0.044 ± 0.002	1.97 ± 0.06	1100 ± 100	1.7 ± 0.1	0.094	0.026	0.28
nVal‐hPhe‐VS	2.6 ± 0.4	2.8 ± 0.9	940 ± 160	2.0 ± 0.1	0.6 ± 0.2	3160 ± 750	2.2 ± 0.1	0.38 ± 0.08	5830 ± 970	3.36	0.54	0.16

a Specificity ratio was calculated based on *k*_inact_/*K*_i_ values; numbers highlighted in bold indicate more than 100‐fold selectivity for the indicated enzymes. Standard error of the global fit for each parameter and inhibitor obtained by fitting *k*_obs_ values (single replicate) to Eqs. 6 or 7 are shown.

Compared to hPhe, P1 nLeu(o‐Bzl) decreases *k*_inact_/*K*_i_ value for DPAP3 by 4‐ to 18‐fold except in the context of a P2 Trp where it increases it by 24‐fold, or a P2 hPro where there is no significant change (Table 4). These differences are mainly due to changes in *K*_i_ rather than *k*_inact_ and likely reflect cooperativity between the S1 and S2 pockets of DPAP3. However, in the case of DPAP1 and CatC, replacement of P1 hPhe with nLeu(o‐Bzl) decreases *K*_i_. Because of this P1‐P2 interdependence, the most potent inhibitors for DPAP3 are either a combination of P2 Tyr(NO_2_) and P1 hPhe, that is, SAK1, or P2 Trp and P1 nLeu(o‐Bzl), resulting in *k*_inact_/*K*_i_ values close to 10^6^ m ^−1^·s^−1^. Importantly, nVal‐hPhe‐VS is as potent as these two inhibitors (*k*_inact_/*K*_i_ = 940 000 m ^−1^·s^−1^), confirming that optimal inhibitors can be designed based on the structure of optimal substrates. These high second order rate constants are mainly driven by low *K*_i_ values (<5 nm). While Trp‐nLeu(o‐Bzl), Igl‐hPhe‐VS and Tyr(NO_2_)‐hPhe‐VS show more than 100‐fold selectivity for DPAP3 over DPAP1, only the latter is selective for DPAP3 compared to CatC (Table 4). On the other hand, nVal‐hPhe‐VS is equally potent for all DPAPs (*k*_inact_/*K*_i _= 1·10^6^, 3.2·10^6^ and 5.8·10^6^ m ^−1^·s^−1^ for DPAP3, DPAP1 and CatC respectively) making it a highly potent but nonselective pan‐DPAP inhibitor

Pro‐hPhe‐VS (SAK2) is 100‐fold more selective towards DPAP1 than DPAP3, but only shows a 10‐fold selectivity for DPAP1 compared to CatC. While replacing the P2 Pro of SAK2 with hPro increases the potency of the inhibitors towards DPAP1, this also results in some loss of specificity (Table 4). Overall, we were able to increase the potency of SAK2 (Pro‐hPhe‐ACC) towards DPAP1 by sevenfold by using the optimal P1 (nLeu(o‐Bzl)) and P2 (hPro) residues identified from the substrate library screen 35, making hPro‐nLeu(o‐Bzl)‐VS the most potent DPAP1 inhibitor identified so far (*k*_inact_/*K*_i _= 3.6·10^6^ m ^−1^·s^−1^) 25, 26, 43. However, this sevenfold increase in inhibitor potency between Pro‐hPhe‐VS and hPro‐nLeu(o‐Bzl)‐VS was much lower than expected based on the 20‐ and 30‐fold increase in substrate turnover reported when replacing P1 hPhe with nLeu(o‐Bzl), and P2 Pro with hPro respectively 35.

### Homology modelling and docking studies

**Figure 5:**
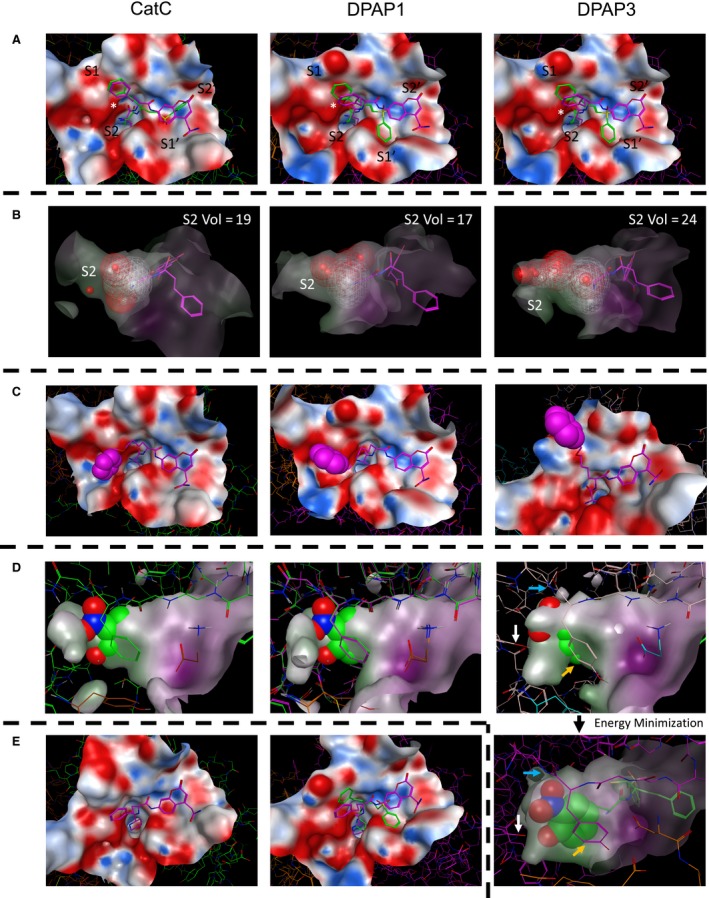
Docking studies on DPAPs. For all panels, the docked structures of compounds into the crystal structure of CatC or the homology models of DPAP1 and DPAP3 are shown on the left, middle and right images respectively. Inhibitors are shown in green, substrates in purple and the surface of the active sites within 6 Å of the ligand as an electrostatic surface. (A) Top views of nVal‐hPhe‐VS and nVal‐hPhe‐ACC docked into the active site of the different DPAPs. The position of the S2 to S2′ pockets is indicated, and that of the exclusion domain N‐terminal Asp is marked with an asterisk. (B) Side view of nVal‐hPhe‐VS docked into the active site of the different DPAPs illustrating the difference in size of the S2 pocket. The dashed volume area defines the volume of the S2 pocket for each DPAP and was calculated by using the ‘Site Finder’ function of MOE. The number on the upper right of each image indicates the calculated relative volume of the S2 pocket. (C) Images illustrating the flexibility of the nLeu(o-Bzl) side chain when docking the nVal‐Leu(o‐Bzl)‐ACC substrate into the different active sites. The benzyl group (spacefill atoms) can reach into different conserved groves distal from the S2 and S1 pockets. (D) Side view of the S2 pocket after modelling the nTyr(NO_2_)‐hPhe‐VS inhibitor into the structures of each DPAP after covalent modification of the catalytic cysteine by the vinyl sulfone group. The surface of the S2 pocket is shown to illustrate steric clashes between the P2 side chain (spacefill atoms) and the S2 pocket. The structure of the inhibitor bound to DPAP3 was further refined by allowing energy minimization of residues within 4.5 Å of the Tyr(NO_2_) side chain (lower right image). Note that in DPAP3, the NO_2_ group might form hydrogen bonds with the amide bond of Ile552 (blue arrows) and with the hydroxylic group of Tyr716 (white arrows) at the bottom of the S2 pocket. We also observed potential stacking interactions between Tyr551 (orange arrows) and the free amine of the inhibitors, as well as hydrophobic interactions with the Tyr(NO_2_) side chain. (E) Docking of hPro‐hPhe‐ACC into the structure of CatC and of hPro‐hPhe‐VS and hPro‐hPhe‐ACC into the model of DPAP1. Note that neither of these compounds could be docked into the DPAP3 model, nor hPro‐hPhe‐VS into the CatC structure.

In order to study the differences in substrate and inhibitor specificity between the different DPAPs, homology models of DPAP1 and DPAP3 were built based on the crystal structure of CatC. Selected compounds were docked into these structures using the MOE (Molecular Operating Environment) software. To visualize differences in the general structure of the active sites, the structures of the pan‐DPAP inhibitor and substrate nVal‐hPhe‐VS and nVal‐hPhe‐ACC were superimposed into the active sites of each DPAP (Fig. 5A). As expected, the hPhe side chain is solvent exposed in the S1 pocket, the free N termini are in close proximity to the carboxylic group of the exclusion domain N‐terminal Asp, and the nVal side chain points into the S2 pocket. The images in Fig. 5A,B clearly show that the DPAP3 S2 pocket is significantly larger than that of DPAP1 or CatC. To better quantify the volume of the S2 pockets, we used our models docked to the nVal‐hPhe‐VS inhibitor and applied the ‘Site Finder’ function in MOE to identify the residues that form the S2 pocket in each protease, and to calculate its volume (Fig. 5B). The relative volumes thus obtained for DPAP1, CatC and DPAP3 are 17, 19 and 24, thus confirming that the S2 pocket of DPAP3 is significantly larger than that of DPAP1 or CatC, and that the CatC S2 pocket is slightly larger than the DPAP1 one. These differences in size explain the specificity of these proteases with DPAP3 being the only one able to accommodate large aromatic P2 residues such as Trp or Tyr(NO_2_), and DPAP1 preferring substrates with relatively small P2 AA such as Val, hAla, Ser or nVal. Similarly to DPAP1, CatC has a preference for small P2 residues but can also accommodate slightly bigger side chains such as Phe, His or 2fa (Fig. 1 and Table 2).

To explain why DPAPs preferentially cleave substrates containing long hydrophobic non‐natural AAs in P1, the structure of nVal‐nLeu(o‐Bzl)‐ACC was docked into the different DPAP structures (Fig. 5C). Although it is difficult to predict how this side chain will bind in the active site of each enzyme given the flexibility of the aliphatic chain, the docked structures indicate that the phenyl group of nLeu(o‐Bzl) might be able to bind beyond the S1 pocket into groves that are not accessible to natural amino acids. Our docking studies identified two potential distal binding sites: one above the S1 pocket (See DPAP3 model in Fig. 5C), the other adjacent to the S2 pocket (DPAP1 and CatC models in Fig. 5C). Of particular interest is the well‐defined grove next to the S2 pocket that is at the interface of the exclusion and catalytic domains. These pockets are present in all DPAPs, which might explain why long hydrophobic residues in P1 make better substrates for this enzyme family.

Finally, we performed docking studies to try to explain why Tyr(NO_2_) and hPro are the preferred P2 residues for DPAP3 and DPAP1 respectively (Fig. 5D,E). For both DPAP1 and CatC, we observed proper docking of hPro‐hPhe‐ACC substrate, with the hPro residue fitting tightly into the S2 pocket, and the secondary amine of hPro pointing towards the N‐terminal Asp side chain of the exclusion domain (Fig. 5E). However, we were unable to obtain any reasonable conformation of this substrate bound to DPAP3. These results reflect the difference in substrate turnover for the three DPAPs: *k*_cat_/*K*_m_ = 320 000 m ^−1^·s^−1^ for DPAP1, 116 000 m ^−1^·s^−1^ for CatC and 300 m ^−1^·s^−1^ for DPAP3. On the other hand, the hPro‐hPhe‐VS inhibitor could only be docked properly into DPAP1, which again might reflect the differences in *k*_inact_/*K*_i_ values: 1 230 000 m ^−1^·s^−1^ for DPAP1 *vs*. 69 000 and 76 000 m ^−1^·s^−1^ for DPAP3 and CatC respectively.

We were not able to dock the Tyr(NO_2_)‐hPhe‐VS compound into any of the DPAP structures. This is especially surprising since this is the most potent DPAP3 inhibitor identified so far. As an alternative, we modelled how this inhibitor would fit into the DPAPs active sites after covalent modification of the catalytic Cys by the vinyl sulfone electrophile (Fig. 5D). In these models, we clearly see that the Tyr(NO_2_) side chain makes very significant steric clashes in the DPAP1 and CatC S2 pockets, but only a few were observed in DPAP3. The steric clashes in DPAP3 could easily be avoided by allowing movement of the side chains forming the S2 pocket as shown in the energy minimized structure of Fig. 5D. This docked structure identified potential interactions between the Tyr(NO_2_) side chain and DPAP3 that can explain why Tyr(NO_2_)‐hPhe‐VS is such a potent DPAP3 inhibitor (*k*_inact_/*K*_i_ = 950 000 m ^−1^·s^−1^). These include hydrogen bonds between the NO_2_ group and the amide bond of Ile552 and the hydroxylic group of Tyr716 at the bottom of the pocket, stacking interactions with Tyr551 and the free amine of the inhibitor and further hydrophobic interactions with Tyr551. This very tight fit within the S2 pocket might also explain why the equivalent substrate Tyr(NO_2_)‐hPhe‐ACC has a relatively low turnover rate given that potential cooperativity in binding between the S2 and S’ pockets might prevent binding of the Tyr(NO_2_) side chain into the S2 pocket. Indeed, the *K*_m_ for Tyr(NO_2_)‐hPhe‐ACC is 9.9 μm while the *K*_i_ for Tyr(NO_2_)‐hPhe‐VS is 2.4 nm. We speculate that the larger size of the substrate ACC group, compared to the inhibitor phenyl group, might prevent proper binding to the Tyr(NO_2_) side chain deep into the S2 pocket.

### Testing inhibitor specificity in live parasites

We then tested the potency and selectivity of our DPAP1‐ and DPAP3‐specific inhibitors in live parasites using the FY01 activity‐based probe (ABP) in a competition assay. ABPs are small molecule reporters of activity that use the catalytic mechanism of the targeted enzyme to covalently modify its active site. A reporter tag, usually a fluorophore or a biotin, allows visualization and quantification of the labelled enzymes in a gel‐based assay 44. FY01 is a cell‐permeable fluorescent ABP that was initially developed for CatC 45 but also labels *Plasmodium* DPAPs and, to a lesser extent, the falcipains 25, 26. Falcipains (FPs) are clan CA cysteine proteases involved in haemoglobin degradation (FP2, FP2′ an FP3) 46 and possibly RBC invasion (FP1) 47, 48. Binding of inhibitors into the active site of any of these cysteine proteases prevents probe labelling resulting in the disappearance of fluorescent bands in a SDS/PAGE gel.

**Figure 6:**
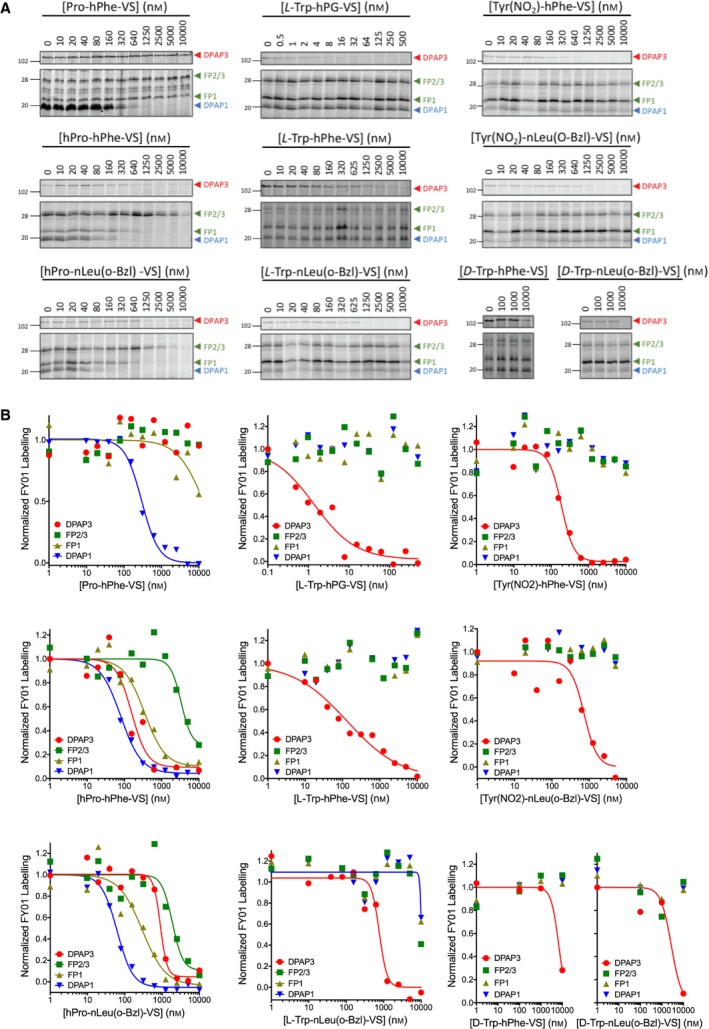
Selective inhibition of DPAP1 or DPAP3 in live parasites. Intact mature schizonts were pretreated for 30 min with increasing concentrations of inhibitor followed by 1 h treatment with FY01. Samples were then run in an SDS/PAGE gel and the in‐gel fluorescence measured using a fluorescence scanner. (A) Representative gel images obtained for each of the inhibitors. Fluorescent bands corresponding to the different cysteine proteases labelled by the probe are indicated with arrowheads (DPAP3, red; FPs, green; DPAP1, blue). (B) The fluorescence intensity of each of the indicated bands was quantified by densitometry using ImageJ, and the fluorescence values normalized to the DMSO control. IC_50_ values are reported in Table 5. Note that control inhibitors containing *D‐*Trp are more than 1000‐fold less potent than those with *L*‐Trp. Three technical replicates were performed; dose responses for a representative replicate are shown.

Live parasites were treated with different concentrations of inhibitor for 30 min, and the residual level of DPAPs and FPs activities were labelled with FY01 and quantified by densitometry (Fig. 6A). Dose response curves are shown in Fig. 6B, and IC_50_ values are reported in Table 5. Inhibitors with a P2 Pro or hPro are equally potent and inhibit DPAP1 at mid nanomolar concentrations. However, P2 Pro makes the inhibitor more selective since it does not target the FPs. Compounds with a P2 Trp or Tyr(NO_2_) are highly specific for DPAP3 in intact parasites. Surprisingly, the inhibitor with homoprolylglycine in P1 (Trp‐hPG‐VS) is by far the most potent DPAP3 inhibitor in intact parasites (IC_50_ = 1.4 nm) despite being five‐ to 100‐fold less potent than Trp‐nLeu(o‐Bzl)‐VS or Trp‐hPhe‐VS against recombinant DPAP3 (see *k*_inact_/*K*_i_ values in Table 4). This suggests that this compound is metabolically more stable and/or that decreasing the hydrophobicity of the P1 residue enhances the cell permeability of the compound. Inhibitors need to cross four membranes to reach DPAP3: the RBC, parasitophorous vacuole and parasite plasma membranes, plus the membrane of the apical organelle where DPAP3 resides 21. (The parasitophorous vacuole is a membrane‐bound structure within which the parasite grows and multiplies isolated from the RBC cytosol.) However, crossing of the parasitophorous vacuole membrane is not likely to be a limiting factor to reach DPAP3 since this membrane is generally permeable to small molecules. It is also likely that the apparent increased potency of Trp‐hPG‐VS in live parasites might be due to its accumulation into the DPAP3 acidic organelle via protonation of its free amine. However, we predict that this lysosomotropic effect likely occurs for all DPAP inhibitors presented here.

**Table 5:** Cysteine proteases IC_50_ values in live parasites

Inhibitor	IC_50,DPAP3_ (nm)	IC_50,DPAP1_ (nm)	IC_50,FP1_ (nm)	IC_50,FP2/3_ (nm)
hPro‐hPhe‐VS	160 ± 50	80 ± 7	350 ± 80	3300 ± 1000
Pro‐hPhe‐VS	> 10 000	275 ± 25	~ 10 000	> 10 000
hPro‐nLeu(o‐Bzl)‐VS	900 ± 100	62 ± 7	300 ± 100	1900 ± 500
Tyr(NO_2_)‐hPhe‐VS	190 ± 20	> 10 000	> 10 000	> 10 000
Tyr(NO_2_)‐nLeu(o‐Bzl)‐VS	730 ± 170	> 10 000	> 10 000	> 10 000
L‐Trp‐hPG‐VS	1.4 ± 0.4	> 10 000	> 10 000	> 10 000
L‐Trp‐hPhe‐VS	130 ± 60	> 10 000	> 10 000	> 10 000
L‐Trp‐nLeu(o‐Bzl)‐VS	760 ± 150	~ 10 000	~ 10 000	~ 10 000
D‐Trp‐hPhe‐VS	6800 ± 1600	> 10 000	> 10 000	> 10 000
D‐Trp‐nLeu(o‐Bzl)‐VS	2700 ± 1600	> 10 000	> 10 000	> 10 000

Technical triplicates were performed for each compound. Standard errors are shown.

## Discussion

This study provides the first characterization of the specificity of DPAP3, a cysteine protease important for efficient invasion of RBCs by the malaria parasite 21. DPAP3 is a highly efficient proteolytic enzyme showing similar *k*_cat_ and *k*_cat_/*K*_m_ values as those measured for DPAP1 or CatC when using optimal substrates (Table 1). In general, DPAP3 has a narrower substrate specificity than either DPAP1 or CatC, and it preferentially cleaves substrates with aliphatic residues at the N terminus. Our study also shows similar P1 substrate specificity across all DPAPs, that is, a strong preference for basic or aromatic residues.

The previously described DPAP1 inhibitor SAK2 (Pro‐hPhe‐VS) shows the greatest specificity for DPAP1 in live parasites. Incorporation of optimal P1 (nLeu(o‐Bzl)) and P2 (hPro) residues identified from the substrate screen improves the potency of DPAP1 inhibitors both *in vitro* and in live parasites, but also results in some loss in specificity (Table 4 and Fig. 6).

DPAP3 was the only DPAP able to cleave some substrates with aromatic P2 residues (Tyr, Phe(Me), Phg), albeit with relatively low turnover rates. This is consistent with the larger volume of the S2 pocket observed in our modelling studies (Fig. 5). Surprisingly, vinyl sulfone inhibitors with P2 aromatic residues are highly specific for DPAP3 and as potent as compounds with optimal P2 residues identified from the substrate screen (Table 4 and Fig. 6). Our docking studies with Tyr(NO_2_)‐hPhe‐VS predict very significant steric clashes in the S2 pocket of DPAP1 and CatC (Fig. 5D), explaining why this inhibitor is more than 1000‐fold specific for DPAP3 (Table 4). We also observed that the Tyr(NO_2_) side chain is able to fully occupy the DPAP3 S2 pocket and form potential hydrogen bond, stacking and hydrophobic interactions with residues in this pocket, which explain the potency of this inhibitor (Fig. 5D). Finally, our computational and experimental studies suggest the presence of cooperativity between the S2 and S’ pockets of DPAP3, which can explain the differences in specificity observed between substrates and inhibitors containing large hydrophobic residues.

Despite being highly potent DPAP1 and/or DPAP3 inhibitors, the vinyl sulfone inhibitors presented here only show antiparasitic activity at mid to high micromolar concentrations 21, 25, 26, probably due to metabolic stability issues. Indeed, we have previously shown that this is the case for Pro‐hPhe‐VS (SAK2), which is not able to sustain target inhibition in live parasites 25. A possible cause for this instability is the presence of multiple aminopeptidases in the malaria parasite that might cleave the amide bond of these compounds 49, thus preventing them from binding into the DPAPs active sites. Nonetheless, this study provides a strong SAR foundation to develop potent nonpeptidic inhibitors able to sustain DPAP inhibition. From a drug development point of view, our SAR studies indicate that inhibitors with short aliphatic P2 residues strongly inhibit both DPAPs (Fig. 4E), and that potent pan‐DPAP inhibitors can be developed. Unfortunately, we did not identify any clear P1 or P2 residue that would discriminate malarial DPAPs from host CatC. Therefore, further studies are required to determine whether differences in specificity in the prime binding pockets can be exploited to develop parasite‐specific inhibitors.

Interestingly, our docking studies with nVal‐nLeu(o‐Bzl)‐ACC identified potential distant binding sites that can only be reached with non‐natural AA and that could explain the DPAPs preference for long hydrophobic residues in P1 (Fig. 5C). Of particular interest is the well‐defined pocket adjacent to the S2 pocket at the interface of the catalytic and exclusion domains that is present in all DPAPs (Fig. 5A). Because DPAPs are the only clan CA proteases with an exclusion domain, designing compounds that bind into this pocket might be a good strategy to prevent off‐target effects against other clan CA endopeptidases. In addition, our homology models suggest that this pocket is much deeper in DPAP1 and DPAP3 than in CatC (Fig. 5A). Therefore, compounds designed to bind deep into this pocket might be specific for *Plasmodium* DPAPs.

While inhibition of host CatC might be a concern when developing DPAP inhibitors as antimalarials, it is important to point out that given the short‐term treatment required for antimalarial therapy (single dose or less than 3 doses in 3 days), we think it is unlikely that inhibition of CatC would lead to adverse side effects. Firstly, highly specific DPAP inhibitors might not be necessary given that a high level (> 95%) of sustained CatC inhibition is required to induce a decrease in the activity of serine proteases activated by CatC 50. Secondly, activation of granule serine proteases by CatC takes place during cell differentiation in the bone marrow, and a decrease in the levels of serine proteases activities in circulating immune cells is only achieved after more than 2 weeks of daily treatment with CatC inhibitors 19. And thirdly, no signs of toxicity were observed in phase I clinical trials when volunteers were treated daily for more than 3 weeks with CatC inhibitors, albeit some on‐target side effects such as plantar and palmar epithelial desquamation were observed in some instances 18, 19. However, these side effects were not observed in volunteers who received a single dose of CatC inhibitor, nor within the first week of daily treatments.

Positional scanning substrate libraries have been successfully used over the last 20 years 30, 31, 32 to determine the specificity of proteases and guide the synthesis of inhibitors. For example, highly potent and specific inhibitors for caspases 51, neutrophil elastase 38, the proteasome 52 or the Zica virus NS2B‐NS3 protease 53, have been developed based on the substrate specificity of proteases. Here, we have also shown that vinyl sulfone inhibitors containing P1 and P2 residues corresponding to optimal DPAP1 or DPAP3 substrates result in extremely potent inhibitors. However, our studies clearly identified differences in amino acid preferences between substrates and inhibitors, especially for DPAP3. This lack of correlation between substrates and inhibitors might not have been more broadly reported in the literature because, in general, either substrate or inhibitor libraries are used to determine the specificity of a protease, but not both. Also, inhibitors are not usually designed based on the structure of nonoptimal substrates, and as indicated above, inhibitors that mimic optimal substrates are generally very potent. That said, there are multiple reasons that can account for discrepancies in specificity between substrates and inhibitors:

First, although the substrate and inhibitor libraries used in this study have equivalent P1 and P2 residues, the structural features that bind into the S’ pockets are quite different. Therefore, if the specificity of a protease shows interdependence between its prime and nonprime binding pockets, it might explain differences in specificity. This is likely the case for DPAPs since we observed a 50‐fold increase in *k*_cat_ between the Phe‐Arg‐ACC and Phe‐Arg‐βNA substrates. These two substrates only differ in the structure of the fluorophore that binds in the S1′ pocket (Table 1). Also, while we observed very good correlation between *k*_cat_/*K*_m_ and *k*_inact_/*K*_i_ of DPAP1 for the P2 substrate and VS library (compounds in Table 2), in the context of a phenyl group in P1′ (inhibitors in Table 4), we observed clear discrepancies between substrates and inhibitors (Fig. 4B).

Second, the position of the electrophilic warhead within the active site might differ from that of the scissile bond in a substrate, especially in terms of distance and orientation relative to the catalytic cysteine. This positioning might be differently affected by changes in the P1 and P2 residues of substrates and inhibitors. Indeed, a recent study on caspases has shown that acyloxymethyl ketone covalent inhibitors might act through a reversible mechanism even if they are designed based on the sequence of optimal substrates 51. However, this is an exception rather than the norm. Also, ABPs designed to profile deubiquitinating proteases by conjugating an electrophile to the C terminal of ubiquitin have been shown to label different subsets of enzymes depending on the warhead used 54.

**Scheme 1:**
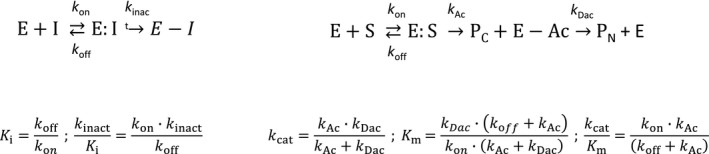
Comparison between irreversible inhibition and substrate turnover kinetic constants. E, I, E:I and E‐I represent free enzyme, inhibitor, inhibitor associated with the enzyme and enzyme covalently modified by the inhibitor respectively. S, E:S, E‐Ac, P_C_ and P_N_ represent the substrate, the Michaelis–Menten enzyme–substrate complex, the acyl intermediate and the C‐ and N‐terminal products of proteolysis respectively; *k*_on_ and *k*_off_ represent the association and dissociation rate constant for substrates or inhibitors; and *k*_A__c_ and *k*_D__ac_ represent the kinetic constants for the formation of the acyl intermediate and its hydrolysis respectively.

Third, substrate turnover by Cys and Ser proteases requires two different chemical steps: First, nucleophilic attack of the peptide bond by the catalytic residue to form the acyl intermediate and release of the C‐terminal product of proteolysis; and second, hydrolysis of the acyl intermediate by an activated water molecule to reconstitute the free enzyme and release the N‐terminal product of the reaction (Scheme 1). Therefore, peptide sequences that are poorly turned over because of a very slow acyl intermediate hydrolysis step might be a good strategy to design covalent inhibitors. For example, a substrate that displaces the catalytic water from the active site upon formation of the acyl intermediate will be very poorly turned over.

And fourth, the reaction mechanism between covalent inhibition and substrate turnover is quite different making *k*_cat_, *K*_m_, and *k*_cat_/*K*_m_ not directly comparable with *k*_inact_, *K*_i_, and *k*_inact_/*K*_i_ (Scheme 1). *k*_cat_, *K*_m_, and *k*_cat_/*K*_m_ are empirical parameters obtained under steady‐state conditions while *k*_inact_, *K*_i_, and *k*_inact_/*K*_i_ are real kinetic and thermodynamic constants that cannot be measured under steady‐state conditions since covalent inhibitors deplete the concentration of free enzyme over time. *k*_cat_ might be equivalent to *k*_inact_ only if *k*_Ac_ << *k*_Dac_, that is, formation of the acyl intermediate is the rate limiting step. *K*_m_ might be equivalent to *K*_i_ only if *k*_Ac_ << *k*_off_ and *k*_Ac_ << *k*_Dac,_ and *k*_cat_/*K*_m_ might be equivalent to *k*_inact_/*K*_i_ only if *k*_off_ >> *k*_Ac_. Although these conditions might be met for certain substrates and inhibitors, the relative magnitudes of all these rate constants depend on the amino acid sequence. For example, detailed presteady‐state kinetics and kinetic isotope effects studies on CatC have shown that the rate limiting step in the turnover of dipeptidic AMC substrates can be either formation of the acyl intermediate, its hydrolysis, or a contribution of both, depending on the nature of the P1 residue 55, 56.

Although several studies have compared the potency of inhibitors to the turnover of equivalent substrates for selected peptide sequences, to the best of our knowledge, this is the first study that systematically compares the potency of a peptide‐based covalent inhibitor library with the turnover efficiency of a substrate library at a specific position. We think that the discrepancies observed here between *k*_cat_/*K*_m_ and *k*_inact_/*K*_i_ might be present in other proteases, but the level of discrepancy will be dependent on the protease studied as well as on the type of substrate and covalent inhibitor that are being compared. We also predict that these discrepancies will be more pronounced if cooperativity exists between the prime and nonprime binding pockets.

Finally, we would like to point out that discrepancies between substrates and inhibitors have been reported in the literature. Some studies on caspases 51, 57 and cathepsins 58 have shown that inhibitors designed based on the structure of specific substrates do not always retain their selectivity. Also, in a few instances, the sequence of an optimal substrate or inhibitor might render the equivalent inhibitor or substrate completely inactive 51. Finally, different N‐terminal capping groups in PS‐SCL of substrates have been shown to result in different amino acid preferences in the S4 pocket of the Zika virus NS2B‐NS3 protease 53, 59. Therefore, if cooperativity exists between P4 and other positions, the influence of the capping group on the S4 pocket specificity might also influence that of other pockets.

Overall, our detailed specificity study on DPAPs indicates that there can be substantial differences in specificity between substrates and covalent inhibitors. Although it is now well established that highly potent inhibitors can be developed based on the structure of optimal substrates, this might sometimes result in some loss of specificity. This study clearly demonstrates that optimal inhibitors with improved specificity can be developed based on the structure of relatively poor substrates.

## Materials and methods

### Reagents

The syntheses of the DPAP substrate library and that of Val‐Arg‐ACC, Phe‐Arg‐ACC, Tyr(NO_2_)‐hPhe‐ACC 41 and (PR)_2_Rho 41 have been previously described. Additional substrates used in this study and were synthesized following previously published methods 35, 60. One gram (0.74 mmol) of Fmoc‐protected Rink Amide resin (Iris Biotech GmbH, Germany) was added to a glass solid‐phase reaction vessel. Next, 5 mL of dichloromethane (DCM) was added and the resin was gently stirred once per 10 minutes for 1 h, then filtered and washed three times with *N,N*‐dimethylformamide (DMF). Fmoc‐protecting group was removed using 20% piperidine in DMF (in three cycles: 5 min, 5 min, and 25 min), filtered each time and rinsed with DMF (six times). A ninhydrin test was performed to confirm resin Fmoc deprotection. Next, 2.5 eq of Fmoc‐ACC‐OH (1.85 mmol, 816 mg) was preactivated with 2.5 eq of HOBt (1.85 mmol, 278 mg) and 2.5 eq of DICI (1.85 mmol, 242 μL) in DMF for 3 min and the mixture was added to the resin. Reaction was gently agitated for 24 h at room temperature. Next, resin was washed five times with DMF and the reaction was repeated using 1.5 eq of the above reagents to improve the yield of ACC coupling. After 24 h of gentle stirring, resin was washed with DMF and Fmoc‐protecting group was removed from ACC with the use of 20% piperidine in DMF (5 min, 5 min, and 25 min), filtered and washed with DMF (six times). Resin was subsequently washed with DCM (3 times) and MeOH (3 times), dried over P_2_O_5_ and divided into eight equal portions (0.09 mmol per portion). Each portion of the H_2_N‐ACC‐resin was placed into the wells of semiautomatic FlexChem solid phase synthesizer cartridge (SciGene, USA). Then, to each well, 2.5 eq of Fmoc‐P1‐OH (0.225 mmol) with 2.5 eq of HATU (0.225 mmol, 86 g), and 2.5 eq of 2,4,6‐collidine (0.225 mmol, 30 μL) in DMF were added. Reactions were carried out for 24 h with gentle agitation of reaction cartridge, followed by washing the resin five times with DMF. P1 coupling reactions were repeated using 1.5 eq of above reagents. P1 Fmoc‐protecting group was removed from each substrate using 20% piperidine in DMF (5 min, 5 min, and 25 min), and the resin was washed six times with DMF. A ninhydrin test was performed to confirm P1 Fmoc deprotection. Next, 2.5 eq Fmoc‐P2‐OH (0.225 mmol) was preactivated with 2.5 eq of HOBt (0.225 mmol, 34 mg) and 2.5 eq of DICI (0.225 mmol, 30 μL) in DMF, added to the cartridge wells containing 1 eq of H_2_N‐P1‐ACC‐resin and gently agitated for 3 hours. A ninhydrin test confirmed the complete P2 coupling. Next, resin was filtered and washed with DMF (six times). Fmoc‐protecting group was removed using 20% piperidine in DMF (5 min, 5 min and 25 min), followed by washing the resin six times with DMF and performing a ninhydrin test. Next, the HN2‐P2‐P1‐ACC‐resin product was washed with DMF (six times), DCM (three times) and MeOH (three times), dried over P_2_O_5_ and cleaved from the resin with a mixture of TFA:TIPS:H2O (v/v/v 95/2.5/2.5). The crude product was purified by HPLC (Waters system), lyophilized and dissolved in DMSO to a final concentration of 20 mm. Each substrate was analysed by analytical HPLC and High Resolution Mass Spectrometry (See Appendix S1).

The syntheses of the vinyl sulfone inhibitor library, SAK2, SAK1, *L‐*WSAK and *D‐*WSAK were also previously described 21, 26. The synthesis of additional vinyl sulfone inhibitors with natural and non‐natural amino acids in the position P1 and P2 is based on a peptide coupling between the activated carboxylic acid of amino acid in the P2, and the unprotected amine of the amino acid P1 bearing the vinyl sulfone. The coupling reaction is followed by the removal of the Boc‐protecting group of P2 to afford the desired inhibitor. The synthesis of the vinyl sulfone was done following previously reported methods 61. Briefly, the carboxylic acid of the amino acid in P1 is coupled with N,O‐Dimethylhydroxylamine to form a Weinreb‐Nahm amide. The amide is reduced to the aldehyde with LiAlH_4_. With the carbonyl at hand, in Horner–Wadsworth–Emmons (HWE) type reaction with diethyl phenylsulfonylmethyl‐phosphonate, the ε‐alkene is formed, which is the nucleophilic trap for the active Cys residue of the protease. The tert‐butyloxycarbonyl‐protecting group is removed in mild acid conditions, and coupled with the activated carboxylic acid as indicated above. Details about the synthesis and structural characterization of these inhibitors and their synthetic intermediates are described in the supplementary methods.

The Phe‐Arg‐βNA and Gly‐Arg‐AMC substrates were purchased from Sigma. Recombinant DPAP3 was expressed in insect cells using the baculovirus system as recently described 21. Bovine CatC was purified to homogeneity from spleen by modification of a method described previously 62, 63.

### Recombinant DPAP3 active site titration

Recombinant DPAP3 was expressed in SF9 insect cells and purified from the culture supernatant by sequential ion exchange, Ni‐NTA and size exclusion chromatography as previously described 21. To accurately determine the concentration of active DPAP3 in our enzyme stocks, we used the FY01 ABP. Because ABPs only react with the active form of an enzyme and covalently modify its active site, in this case the catalytic Cys, they can be used to perform accurate active site titrations.

**Figure 7:**
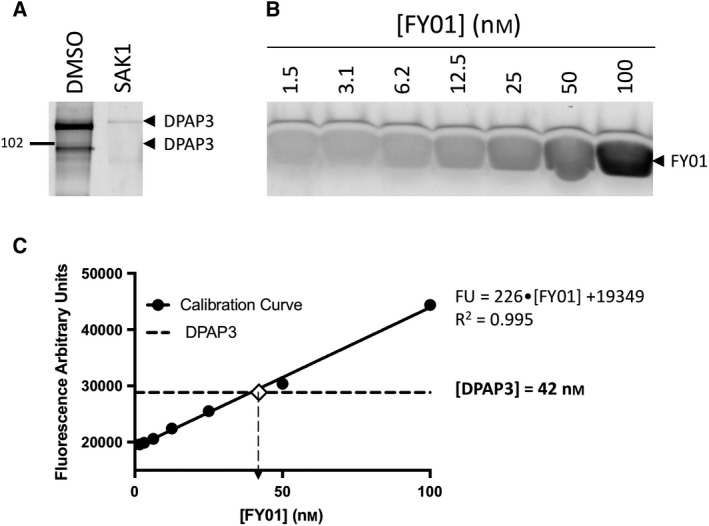
Active site titration of DPAP3 using activity‐based probes. (A) Labelling of purified rDPAP3 by FY01 in the presence or absence of SAK1. Our stock of rDPAP3 was diluted 20‐fold in assay buffer, treated with DMSO or 1 μm SAK1 for 30 min, and labelled with FY01 for 1 h. Samples were run on an SDS/PAGE gel and DPAP3 labelling measured using a flatbed fluorescence scanner. (B) Calibration curve of free probe measured on the same SDS/PAGE gel as the one shown in A. (C) The fluorescence signal for rDPAP3 labelling and free probe was quantified using ImageJ, and the concentration of labelled rDPAP3 calculated based on the calibration curve.

Our stock of DPAP3 was diluted 20‐fold in assay buffer (100 mm sodium acetate, 100 mm NaCl, 5 mm MgCl_2_, 1 mm EDTA, 0.1% CHAPS, and 5 mm DTT at pH 6), pretreated for 30 min with DMSO or 1 μm SAK1 (Tyr(NO_2_)‐hPhe‐VS), and DPAP3 labelled with 1 μm FY01 for 1 h at RT. These samples were run on a SDS/PAGE gel along with a serial dilution of free probe (1.5–100 nm). In‐gel fluorescence was measured using a Bio‐Rad PharosFX flat‐bed scanner and the intensity of the fluorescent bands quantified using ImageJ. The fluorescent signal from the free probe was used as a calibration curve and compared to the difference in signal between the DMSO and SAK1‐treated DPAP3 (Fig. 7). Using this method, we determined that our DPAP3 stock contained 840 nm of active protease.

### Substrate turnover assay

The substrate library was screened in triplicate at 1 μm substrate and 1 nm DPAP3 in assay buffer. Substrate turnover was measured over 30 min at RT using a SpectraMax M5e plate reader (Molecular Devices): λ_ex_ = 355 nm, λ_em_ = 460 nm, emission filter 455 nm, for ACC or AMC (7‐amino‐4‐methylcoumarin) substrates; λ_ex _= 315 nm, λ_em_ = 355 nm, emission filter 420 nm, for Phe‐Arg‐βNA; and λ_ex _= 492 nm, λ_em_ = 523 nm, emission filter 520 nm for (PR)_2_Rho. Calibration curves of free βNA (β‐napthylamide) and ACC (0‐500 nm) under the same assay conditions were performed to convert the turnover rate measured as fluorescent units per second into moles per second. To determine *k*_cat_ and *K*_m_ values, substrate turnover was measured at different substrate concentrations in assay buffer using 1 nm of DPAP3 or 1 nm of bovine CatC. The initial velocities were then fitted with Prism to the Michaelis–Menten Eqs. 3 or 4 to obtain accurate *k*_cat_ and *K*_m_ or *k*_cat_/*K*_m_ values respectively. A minimum of three replicates were performed for each substrate.

(3) $$V_{i}=\frac{k_{\mathrm{cat}}\cdot Et\cdot \left[S\right]}{K_{m}+\left[S\right]}$$

(4) $$V_{i}=\frac{\left(k_{\mathrm{cat}}/K_{m}\right)\cdot Et\cdot \left[S\right]}{1+\frac{\left[S\right]}{K_{m}}}$$

*V*_i_ is the initial velocity and *Et* is the total concentration of active protease.

### Irreversible inhibition assay

To determine the inhibition constants of vinyl sulfone inhibitors against DPAP3, 2.2 μm of Met‐nLeu(o‐Bzl)‐ACC (0.25 x *K*_m_) was mixed with increasing concentrations of inhibitor in assay buffer, and the turnover rate was measured over 40 min at RT after addition of 0.2 nm of DPAP3. The data were analysed according to the irreversible inhibition model shown in Eqn. 1. First, the progress curves (fluorescent units vs. time) were fitted to Eqn. 5, where F is the measured fluorescence at time *t*,* F*_0_ the initial fluorescence, *V*_0_ the initial turnover rate, and *k*_obs_ the observed second order rate constant of inhibition measured at each inhibitor concentration 64.

(5) $$\left(F-F_{0}\right)=V_{0}\frac{1-e^{k_{\mathrm{obs}}\cdot t}}{k_{\mathrm{obs}}}$$

Second, *k*_obs_ values were fitted to Eqs. 6 or 7 to obtain the *k*_inact_ and *K*_i_ or *k*_inact_/*K*_i_ values respectively.

(6) $$k_{\mathrm{obs}}=\frac{k_{\mathrm{inact}}\cdot \left[\text{INH}\right]}{K_{i}+\left[\text{INH}\right]}$$

(7) $$k_{\mathrm{obs}}=\frac{\left(k_{\mathrm{inact}}/K_{i}\right)\cdot \left[\text{INH}\right]}{1+\frac{\left[\text{INH}\right]}{K_{i}}}$$

Inhibition constants for DPAP1 and CatC were determined in a similar way but using 0.25 x *K*_m_ concentrations of the Pro‐Arg‐AMC (20 μm) and Gly‐Arg‐AMC (15 μm) respectively. Inhibition of DPAP1 was directly measured in parasite lysates diluted 100‐fold in assay buffer using the DPAP1‐selective substrate Pro‐Arg‐AMC 42; 0.2 nm of CatC was used for these inhibition studies. Note that because DPAP1 inhibition was directly measured in parasite lysates, inhibitors might have been partially inactivated through the action of aminopeptidases present in the lysates during the course of the assay. Therefore, the potency of the inhibitors might have been underestimated. However, for all inhibitors the kinetics of DPAP1 inhibition is consistent with our inhibition model (Eqn. 1) indicating that time‐dependent degradation of the inhibitor is likely to be negligible.

### Homology modelling and docking studies

Homology models for DPAP1 and DPAP3 were built based on the crystal structure of CatC (PDB:1JQP), and selected inhibitors and substrates were docked into the CatC structure and DPAP1 and DPAP3 models. Phyre was used to build the initial alignment of human CatC, DPAP1 and DPAP3 with PSIRED secondary structure prediction and manual adjustments of the alignment to remove insertion sequence not present in the CatC structure. Alignment used to build the DPAP1 and DPAP3 models showed 33% and 30% sequence identity and 50% and 49% similarity to CatC respectively. MOE.2016.08 was used to build the homology models of DPAP1 and DPAP3 from this alignment. Ten models were built with Protonate3D and coarse minimization applied to the final model. Docking was carried out with Gold v5.6.2. The docking region was defined as a 10 Å radius from the catalytic cysteine. Energy minimization was only applied to the small molecules, not to the enzyme backbone or side chains. Default/automatic settings were employed with GoldScore selected for the scoring function and the top 20 poses saved. Each pose was visually inspected for suitability. The volume of the S2 pocket was calculated based on the docked structures of the nVal‐hPhe‐VS inhibitor. We use the ‘Site Finder’ function in MOE to identify a binding pocket within 9 Å of the δ carbon of the nVal side chain and to calculate a relative volume of the S2 pocket for each of the DPAPs.

### Measurement of inhibitor specificity in live parasites

Anonymized human blood used to culture *P. falciparum* was sourced ethically from the United Kingdom National Health Service Blood and Transplant (NHSBT) Special Health Authority in accordance with the United Kingdom Human Tissue Act, which conforms to the Declaration of Helsinki. Blood was obtained with informed consent of the donors and for research purposes only.

Inhibition of *Plasmodium* DPAPs and the falcipains in intact parasites was measured using the FY01 ABP in competition assays as previously described 25, 26. Because DPAP3 is maximally expressed in very mature schizonts 6, labelling was performed after treating parasites with 1 μm of ‘Compound 2′, a cGMP‐dependent protein kinase inhibitor that arrests parasite development 15‐30 min before they egress from the infected RBC 65. For each sample, 5 μL of percoll‐purified schizonts was diluted in 45 μL of RPMI (Gibco), pretreated for 30 min with a dose response of inhibitor, and labelled for 1 h with 1 μm FY01. Samples were then boiled for 10 min in loading buffer and run on a 12% SDS/PAGE gel. Fluorescently labelled proteases in the gel were detected using a Bio‐Rad PharosFX flatbed fluorescence scanner.

## Conflict of interest

The authors declare no conflict of interest.

## Author contributions

ED planned, performed and managed the experiments, analysed the data and wrote the manuscript. LEV, LK, and CL performed the experiments. MIS, FY, and SAK, synthesized inhibitors under the supervision of MB, and MP and KG substrates under the supervision of MD, MH and MM contributed reagents. CWM, NN and DJH were involved in building the homology models and performing docking studies.

## Supporting information


**Fig. S1.** Michaelis–Menten fits for DPAP3.
**Fig. S2.** Representative irreversible inhibition fits.
**Fig. S3.** Michaelis–Menten fits for CatC.
**Appendix S1.** Synthesis and characterization of inhibitors and substrates.
