# Supplementary material for: Characterization of P. falciparum dipeptidyl aminopeptidase 3 specificity identifies differences in amino acid preferences between peptide‐based substrates and covalent inhibitors
Source: FEBS J. 2019 Jun 24;286(20):3998–4023. doi: 10.1111/febs.14953 (PMC6851853; doi:10.1111/febs.14953)

FEBS PRESS
science publishing by scientists
WILEY
The FEBS Journal
www.febsjournal.org

Characterization of *P. falciparum* dipeptidyl aminopeptidase 3 specificity identifies differences in amino acid preferences between peptide-based substrates and covalent inhibitors

Laura E.de Vries, Mateo I. Sanchez, Katarzyna Groborz, Laurie Kuppens, Marcin Poreba, Christine Lehmann, Neysa Nevins, Chrislaine Withers-Martinez, David J. Hirst, Fang Yuan, Shirin Arastu-Kapur, Martin Horn, Michael Mares, Matthew Bogyo, Marcin Drag and Edgar Deu

DOI: 10.1111/febs.14953

SUPPLEMENTARY MATERIALS

Characterization of *P. falciparum* dipeptidyl aminopeptidase 3 specificity identifies differences in amino acid preferences between peptide-based substrates and covalent inhibitors.

Laura E. de Vries[1,§], Mateo I. Sanchez[2,§], Katarzyna Groborz[3,§], Laurie Kuppens[4,§], Marcin Poreba[3], Christine Lehmann[4], Neysa Nevins[5], Chrislaine Whiters-Marinez[6], David J. Hirst[7], Fang Yuan[8], Shirin Arastu-Kapur[8], Martin Horn[9], Michael Mares[9], Matthew Bogyo[8], and Edgar Deu[4,§,*] .

[1]Department of Medical Microbiology, Radboud University Medical Center, Nijmegen, Netherlands.

[2]Department of Genetics, Stanford School of Medicine, Stanford, California, United States.

[3]Division of Bioorganic Chemistry, Faculty of Chemistry, Wroclaw University of Technology, Wroclaw, Poland.

[4]Chemical Biology Approaches to Malaria Laboratory, The Francis Crick Institute, London, England, United Kingdom.

[5]Computational Sciences, GlaxoSmithKline, Collegeville, United States.

[6]Malaria Biochemistry, The Francis Crick Institute, London, England, United Kingdom.

[7]Crick-GSK Biomedical Linklabs, GlaxoSmithKline, Stevenage, United Kingdom.

[8]Department of Pathology, Stanford University School of Medicine, Stanford, United States.

[9]Institute of Organic Chemistry and Biochemistry, Czech Academy of Sciences, Prague, Czech Republic.

[§] These authors contributed equally to this work.

*Corresponding author:

e-mail: edgar.deu@crick.ac.uk; Tel: +44 (0) 20 3796 1412

Supplementary Results

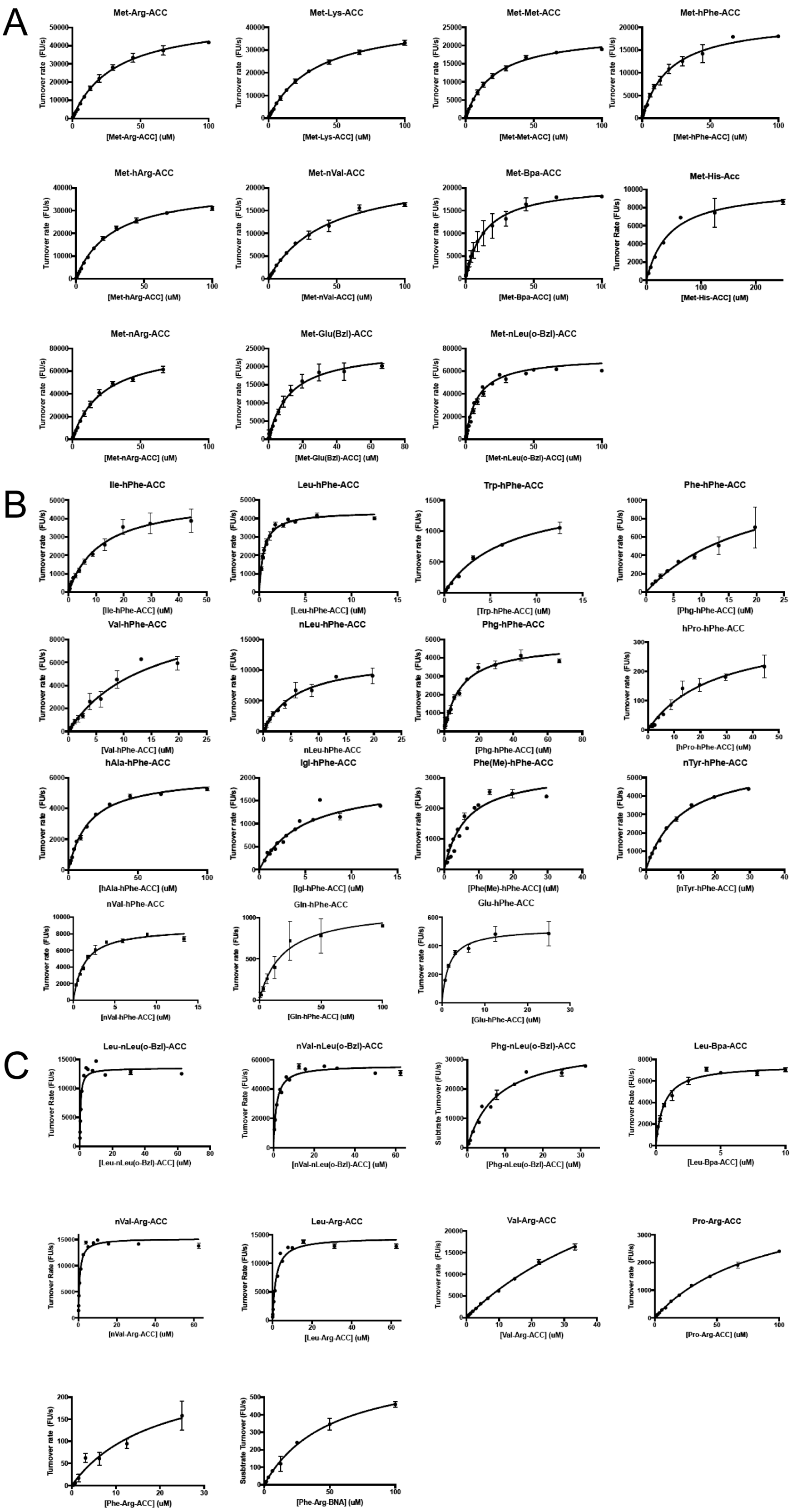

Figure S1. Michaelis Menten fits for DPAP3. The turnover rate of the indicated substrates was measured at different concentrations and 1 nM DPAP3 in assay buffer. Data was fitted in Prism to a Michaelis Menten model. K_m, k_{cat}, and k_{cat}/K_m values are reported in Table 1. (**A**) Substrates with P2 Met. (**B**) Substrates with P1 hPhe. (**C**) Substrates with selected P1 and P2 residues. Error bars represent standard errors (N=3-10 depending on the substrate. See Table 1)

P2: Ala; P1: hPhe

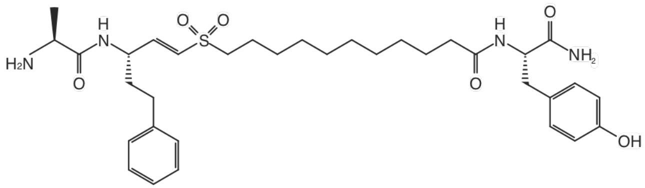

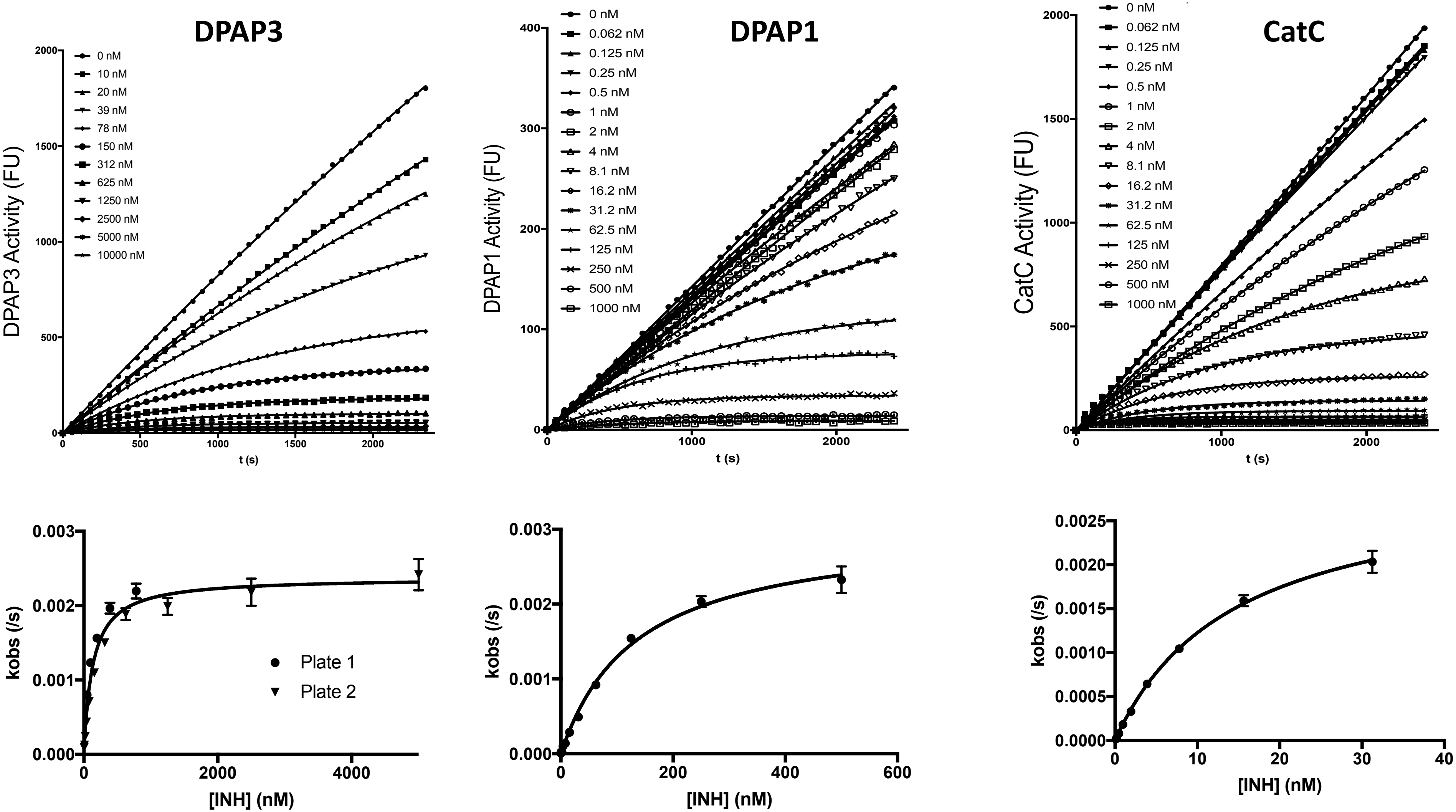

DPAP3

k_{inact} = 0.0024 ± 0.0001 s^{-1}

K_i = 88 ± 13 nM

k_{inact}/K_i = 27,000 ± 3,000 $M^{-1}s^{-1}$

DPAP1

k_{inact} = 0.00303 ± 0.00009 s^{-1}

K_i = 90 ± 7 nM

k_{inact}/K_i = 34,000 ± 2,000 $M^{-1}s^{-1}$

CatC

k_{inact} = 0.00300 ± 0.00007 s^{-1}

K_i = 9.7 ± 0.4 nM

k_{inact}/K_i = 310,000 ± 8,000 $M^{-1}s^{-1}$

P2: hPro; P1: nLeu(oBzl)

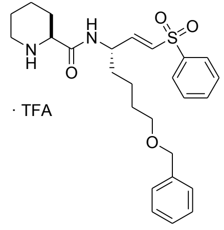

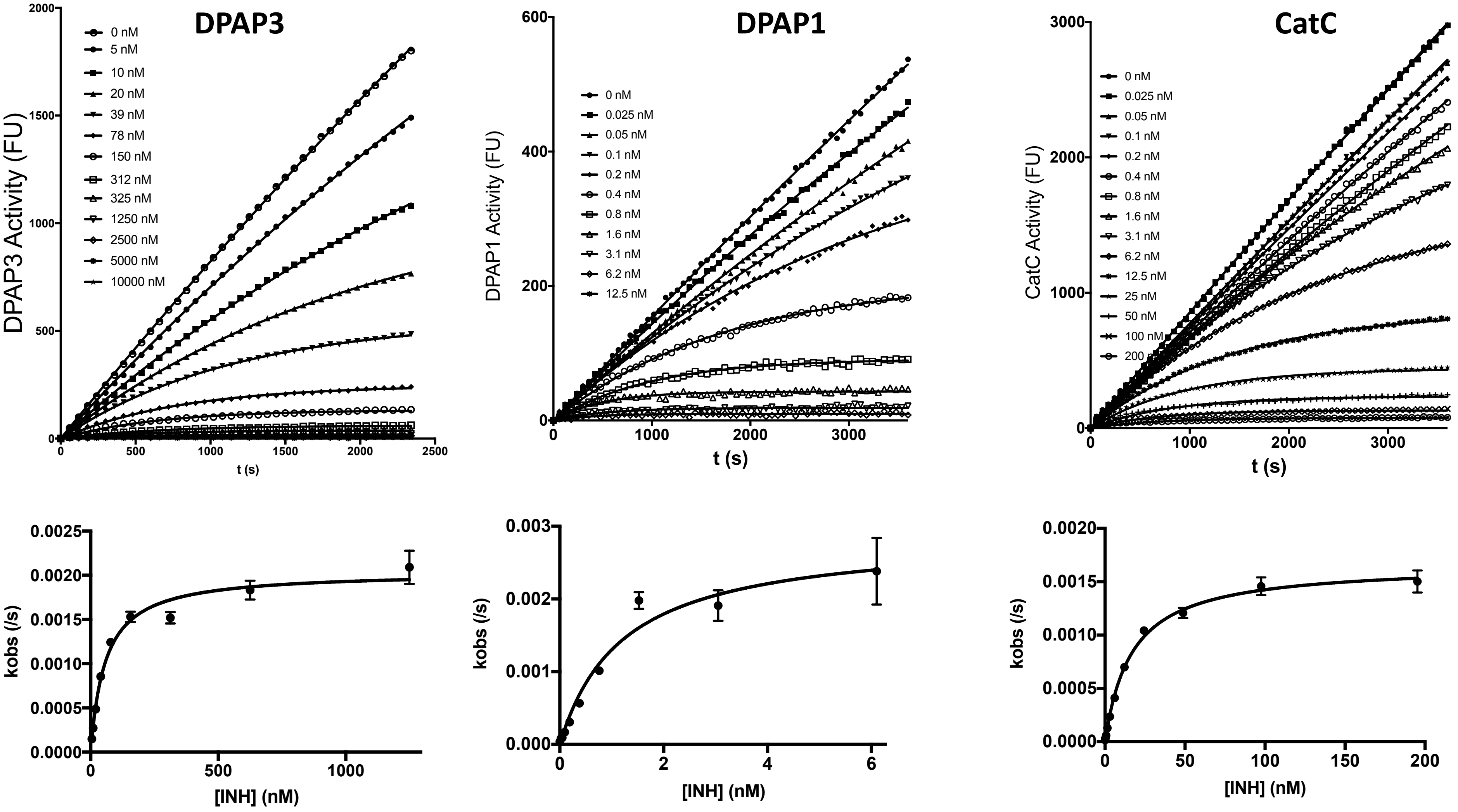

k_{inact} = 0.0020 ± 0.0001 s^{-1}
K_i = 39 ± 6 nM
k_{inact}/K_i = 53,000 ± 6,500 $M^{-1}s^{-1}$

k_{inact} = 0.0029 ± 0.0003 s^{-1}
K_i = 0.8 ± 0.2 nM
k_{inact}/K_i = 3,560,000 ± 150,000 $M^{-1}s^{-1}$

k_{inact} = 0.00167 ± 0.00003 s^{-1}
K_i = 11.2 ± 0.6 nM
k_{inact}/K_i = 150,000 ± 6,000 $M^{-1}s^{-1}$

P2: Tyr(NO_2); P1: hPhe

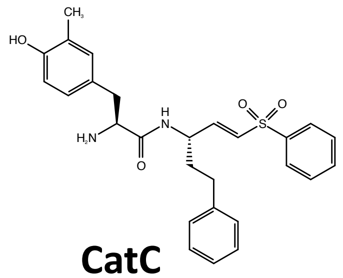

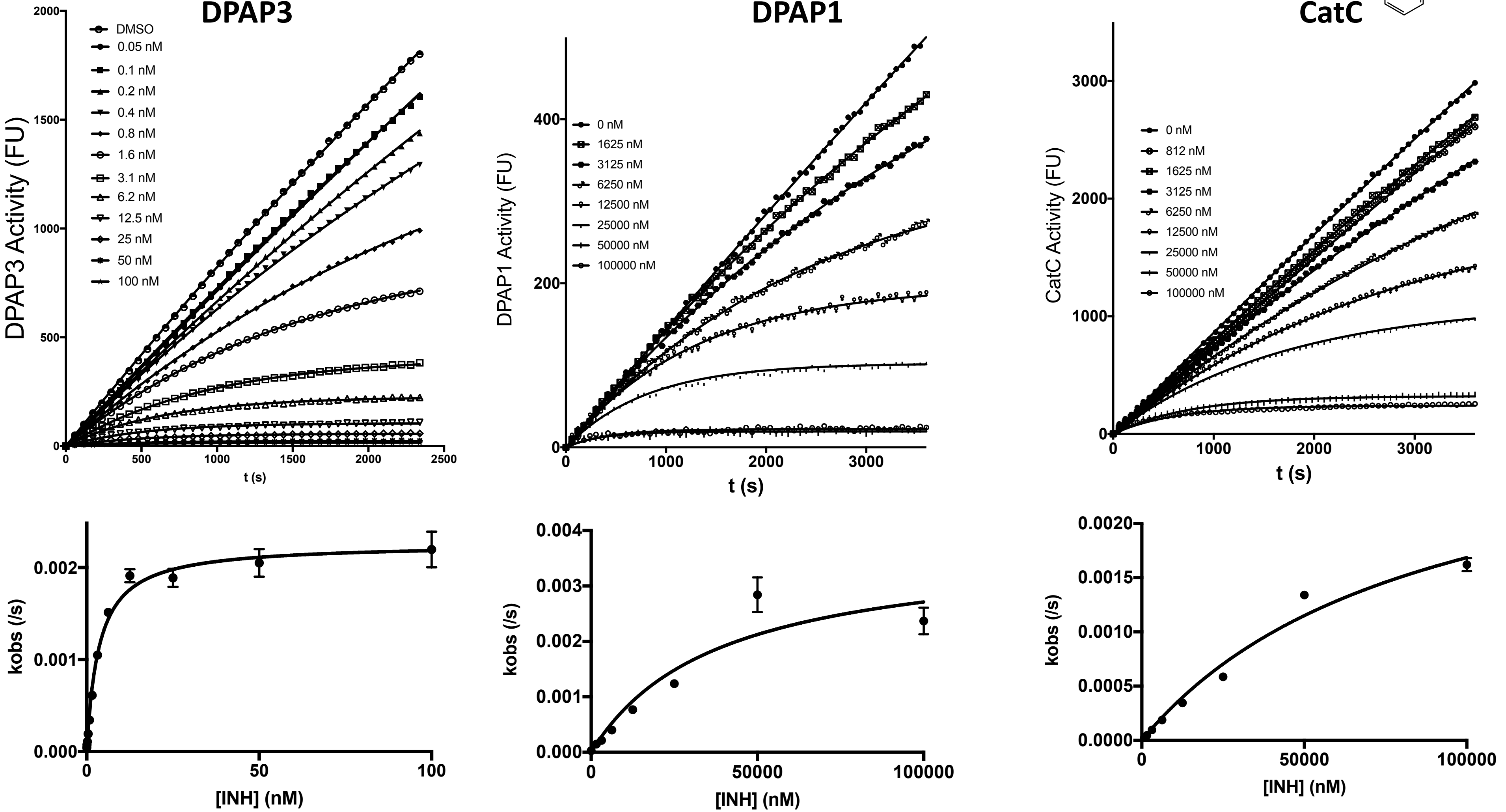

DPAP3	DPAP1	CatC
k_{inact} = 0.00226 ± 0.00005 s^{-1}	k_{inact} = 0.0038 ± 0.001 s^{-1}	k_{inact} = 0.0032 ± 0.0006 s^{-1}
K_i = 2.4 ± 0.2 nM	K_i = 25,000 ± 1,400 nM	K_i = 58,000 ± 20,000 nM
k_{inact}/K_i = 950,000 ± 74,000 $M^{-1}s^{-1}$	k_{inact}/K_i = 150 ± 50 $M^{-1}s^{-1}$	k_{inact}/K_i = 54 ± 8 $M^{-1}s^{-1}$

Figure S2. Representative irreversible inhibition fits. Attached as a separate file. Substrate turnover by rDPAP3 (left row), DPAP1 (middle row) or CatC (right row) was measured for at lest 40 min at different inhibitor concentrations. Representative fits for an inhibitor of the VS library (P2 = Ala) and for the most potent inhibitors of DPAP1 (hPro-nLeu(oBzl)-VS) and DPAP3 (Tyr(NO_2)-hPhe-VS) are shown. The reaction progress curves (FU vs time) at each inhibitor concentration were fitted to Eq. 5 to obtain k_{obs} values (Top graphs). These values were then fitted to Eqs. 6 and 7 as a function of inhibitor concentration to obtain k_{inact}, K_i, and k_{inact}/K_i.

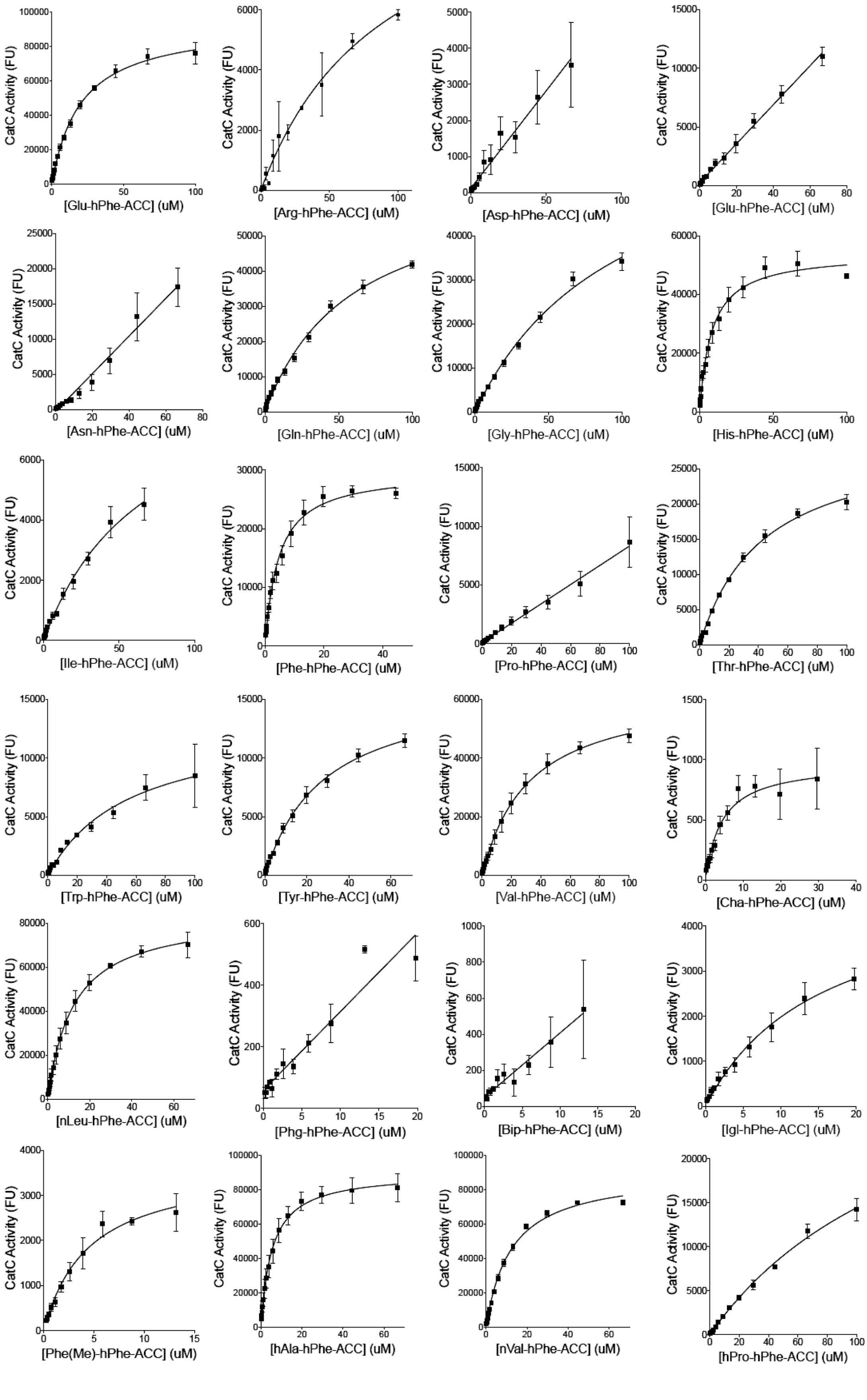

Figure S3. Michaelis Menten fits for CatC. The turnover rate of the indicate substrates was measured at different concentrations and 1 nM CatC in assay buffer. Data was fitted in Prism to a Michaelis Menten model. K_m, k_{cat}, and k_{cat}/K_m values are reported in Table 3.

Supplementary Materials and Methods

High resolution mass spectra of DPAP fluorogenic substrates.

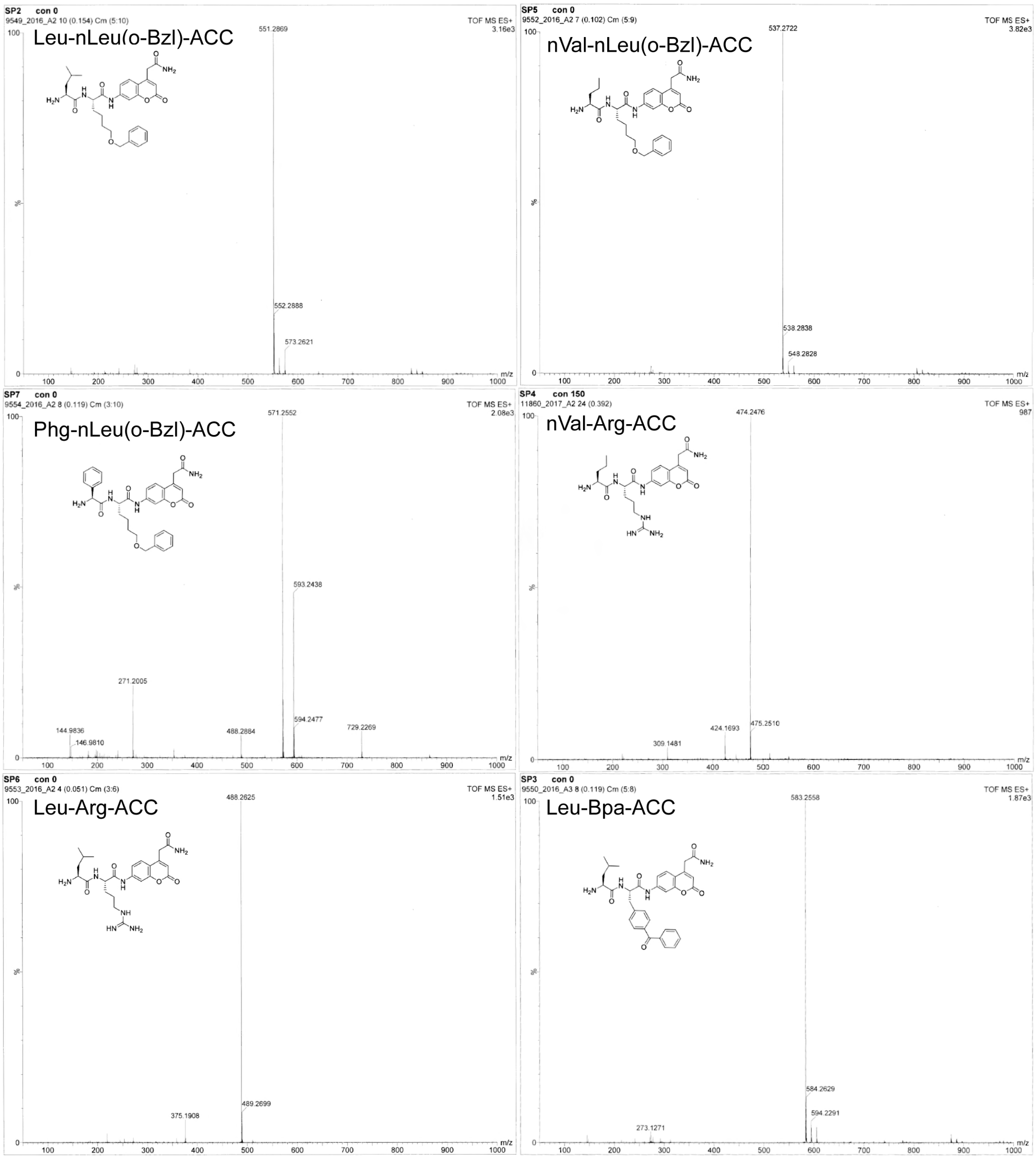

Synthesis of vinyl sulfone inhibitors.

***tert*-Butyl (S)-1-(N-methoxy-N-methylcarbamoyl)pent-4-ynylcarbamate** Lm1msed13

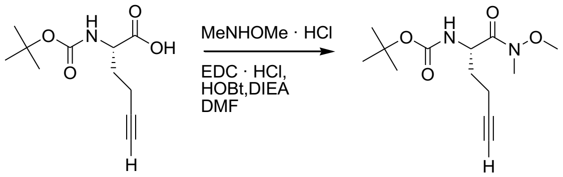

To a solution of (S)-2-(Boc-amino)-5-hexynoic acid (0.6 g, 2.64 mmol) in dry DMF (14 mL) at 0 °C was added EDC (607 mg, 3.17 mmol), HOBt (427 mg, 3.17 mmol), N,O-dimethylhydroxylamine hydrochloride (308 mg, 3.17 mmol) and DIPEA (0.7 mL, 7.92 mmol). The reaction was stirred at rt for 12 h. The resulting solution was evaporated in vacuo to give a light yellow oil, which was diluted with aqueous NH_4Cl (10 wt%) and extracted with EtOAc (3 x 50 mL). The combined organic extracts were dried over Na_2SO_4, filtered and concentrated in vacuo. Purification by flash column chromatography (silica gel; using 20% EtOAc in hexanes) provided the protected product *tert*-butyl (S)-1-(N-methoxy-N-methylcarbamoyl)pent-4-ynylcarbamate as a white foam (0.64 g, 2.37 mmol, 90%).

^{1}H NMR (*DCCl$_3$ δ*):□1.41 (s, 9H) 1.72 (td, J = 20.9, 6.9 Hz, 2H), 1.96 (t, J = 2.6 Hz, 1H), 2.27 (dt, J = 7.2, 7.0, 2.5 Hz, 2H), 3.19 (s, 3H), 3.76 (s, 3H), 4.73 (dt, J = 8.7, 4.4 Hz, 1H), 5.21 (d, J = 8.1 Hz, 1H).

^{13}C NMR (*DCCl$_3$ δ*): 15.0 (CH_2), 28.3 (CH_3), 32.0 (CH_2), 32.2 (CH_3), 49.8 (CH), 61.6 (CH_3), 68.7 (CH), 79.7 (C), 83.2 (C), 155.4 (C), 172.4 (C).

ESI-MS: $[M+H]^+$ calcd. for $C_{13}H_{22}N_2O_4Na$ = 293.1772 found 293.1478. (M.W. 270.3248)

***tert*-Butyl (S)-1-formylpent-4-ynylcarbamate** Lm1msed14

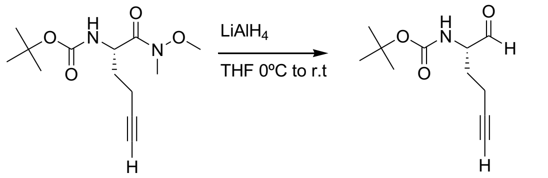

To a solution of *tert*-Butyl (S)-1-(N-methoxy-N-methylcarbamoyl)pent-4-ynylcarbamate (0.62 g, 2.3 mmol) in dry THF (23 mL) at 0 °C was added $LiAlH_4$ (0.1 g, 2.76 mmol) over 10 min, with vigorous stirring. The mixture was stirred for an additional 20 min at 0 °C, whereupon cold water was carefully added until effervescence ceased. A cold HCl solution (1 M) was added to break up the gelatinous emulsion until pH 6~7. Upon dilution with H_2O (150 mL) and extraction with EtOAc (3 x 75 mL), the combined organic extracts dried over Na_2SO_4, filtered and concentrated in vacuo. Purification by flash column chromatography (silica gel; using 30% EtOAc in hexanes) provided the product *tert*-Butyl (S)-1-formylpent-4-ynylcarbamate as a white solid (0.44 g, 2.08, 90%).

^{1}H NMR (*$DCCl_3$ δ*): 1.44 (s, 9H), 1.85 (dt, *J* = 14.2, 14.0, 7.1 Hz, 2H), 2.01 (t, *J* = 2.6 Hz, 1H), 2.32 (dt, *J* = 6.7, 2.5 Hz, 2H), 4.29 (bs, 1H), 5.21 (bs, 1H), 9.64 (s, 1H).

^{13}C NMR (*$DCCl_3$ δ*): 14.5 (CH_2), 27.9 (CH_2), 28.1 (CH_3), 59.0 (CH), 70.0 (CH), 80.2 (C), 82.5 (C), 155.3 (C), 199.0 (CH)

ESI-MS: $[M+H]^+$ calcd. for $C_{11}H_{17}NO_3Na$ = 234.1101 found 234.1105. (M.W. 211.2576)

tert-Butyl (S,E)-1-(phenylsulfonyl)hept-1-en-6-yn-3-ylcarbamate

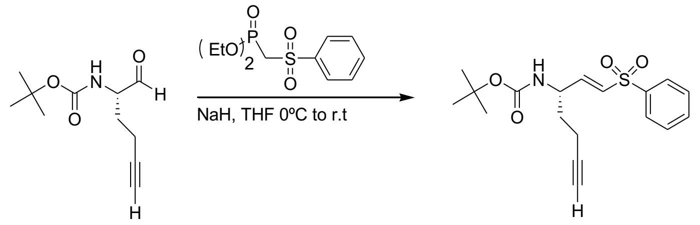

To a cooled (0°C) suspension of hexane-washed NaH (60% in mineral oil; 0.1 g, 2.38 mmol) in dry THF (10 mL) was added drop-wise diethyl[benzenesulfonyl)methyl]phosphonate (0.64 g, 2.18 mmol) in dry THF (10 mL) via syringe. The mixture was stirred for an additional 30 min at 0 °C and *tert*-butyl (S)-1-formylpent-4-ynylcarbamate (0.42 g, 2.0 mmol) in dry THF (10 mL) was added drop-wise. The stirring was continued for 1 h, before a cold 10 wt% NH_4Cl solution was added to break up the gelatinous emulsion until pH 6~7. The solution was concentrated in vacuo, diluted with water (100 mL) and extracted with EtOAc (3 x 75 mL). The combined organic extracts were dried over Na_2SO_4, filtered and concentrated under vacuum. Purification by flash column chromatography (silica gel; using 40% EtOAc in hexanes) provided the product *tert*-butyl (S,E)-1-(phenylsulfonyl)hept-1-en-6-yn-3-ylcarbamate as a white foam (0.46 g, 1.30 mmol, 65%).

^{1}H NMR (*$DCCl_3$ δ*): 1.37 (s, 9H), 1.80 (ddd, *J* = 21.5, 13.8, 6.9 Hz, 2H), 2.00 (t, *J* = 2.6 Hz, 1H), 2.27 (dt, *J* = 6.9, 2.5 Hz, 2H), 4.47 (bs, 1H), 4.69 (d, *J* = 8.6 Hz, 1H), 6.46 (dd, *J* = 15.0, 1.5 Hz, 1H), 6.89 (dd, *J* = 15.0, 5.0 Hz, 1H), 7.50-7.61 (m, 3H) 7.87 (d, *J* = 7.0 Hz, 2H).

^{13}C NMR (*$DCCl_3$ δ*): 15.1 (CH_2), 28.2 (CH_3), 32.7 (CH_2), 50.4 (CH), 69.9 (CH), 80.2 (C), 82.4 (C), 127.6 (CH), 129.2 (CH), 130.9 (CH), 133.4 (CH), 140.1 (C), 145.5 (CH), 154.8 (C).

ESI-MS: $[M+H]^+$ calcd. for $C_{18}H_{23}NO_4Na$ = 372.1240 found 372.1254. (M.W. 349.4445)

(S,E)-1-(phenylsulfonyl)hept-1-en-6-yn-3-amine

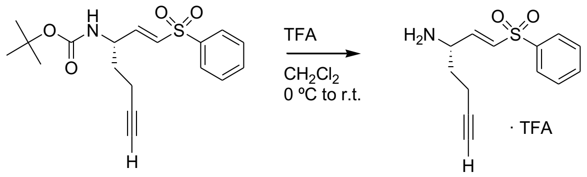

^{1}H NMR (*MeOD-d$_4$ δ*): 1.90-2.07 (m, 2H), 2.20-2.31 (m, 2H), 2.40 (t, J = 2.6 Hz, 1H), 4.14 (dt, J = 7.9, 6.0 Hz, 1H), 6.88 (dd, J = 15.2, 7.4 Hz, 1H), 7.02 (d, J = 15.2 Hz, 1H), 7.61-7.74 (m, 3H), 7.92-7.95 (m, 2H).

^{13}C NMR (*MeOD-d$_4$ δ*): 15.3 (CH_2), 32.1 (CH_2), 51.6 (CH), 72.0 (CH), 82.1 (C), 129.1 (CH), 130.8 (CH), 135.3 (CH), 137.1 (CH), 139.9 (CH), 140.8 (C), 162 (q, C, TFA).

ESI-MS: $[M+H]^+$ calcd. for $C_{13}H_{16}NO_2S$ = 250.0896 found 250.0890. (M.W. 363.3520)

(2S)-2-Amino-3-(1H-indol-3-yl)-N-((S,E)-1-(phenylsulfonyl)hept-1-en-6-yn-3-yl)propanamide (Trp-hPG-VS)

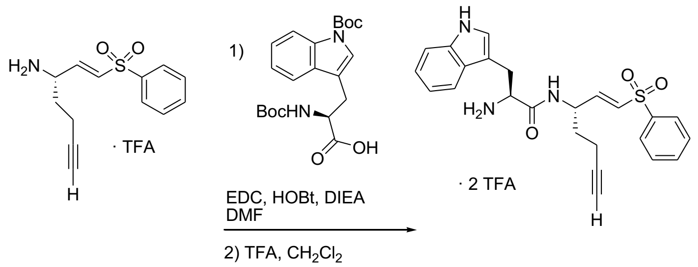

^{1}H NMR (*MeOD$_4$ δ*): 1.73-1.86 (m, 2H), 2.21 (dt, *J* = 7.2, 2.6 Hz, 2H),2.28 (bs, 1H), 3.20-3.35 (m, 2H, partial overlapping with MeOD signal), 4.12 (dd, *J* = 7.80, 7.15 Hz, 1H), 4.70 (dd, *J* = 13.3, 4.8 Hz, 1H), 6.15 (dd, *J* = 15.1, 1.5 Hz, 1H), 6.78 (dd, *J* = 15.1, 5.4 Hz, 1H), 7.05-7.08 (m, 1H), 7.14 (bs, 2H), 7.43 (d, *J* = 8.2 Hz, 1H), 7.57-7.64 (m, 3H), 7.67-7.70 (m, 1H), 7.84 (dd, *J* = 8.4, 1.2 Hz, 2H).

^{13}C NMR (*MeOD$_4$ δ*): 15.2 (CH_2), 28.7 (CH_2), 32.6 (CH_2), 50.2 (CH), 55.1 (CH), 70.7 (CH), 83.3 (C), 108.0 (C), 122.4 (CH), 118.8 (CH), 120.1 (CH), 122.7 (CH), 125.2 (CH), 128.3 (C), 128.5 (CH), 130.3 (CH), 132.2 (CH), 134.6 (CH), 138.2 (C), 141.6 (C), 145.9 (CH). 169.9 (C).

ESI-MS: $[M+H]^+$ calcd. for $C_{24}H_{26}N_3O_3S$ = 436.1689 found 436.1690. (M.W. 663.5853).

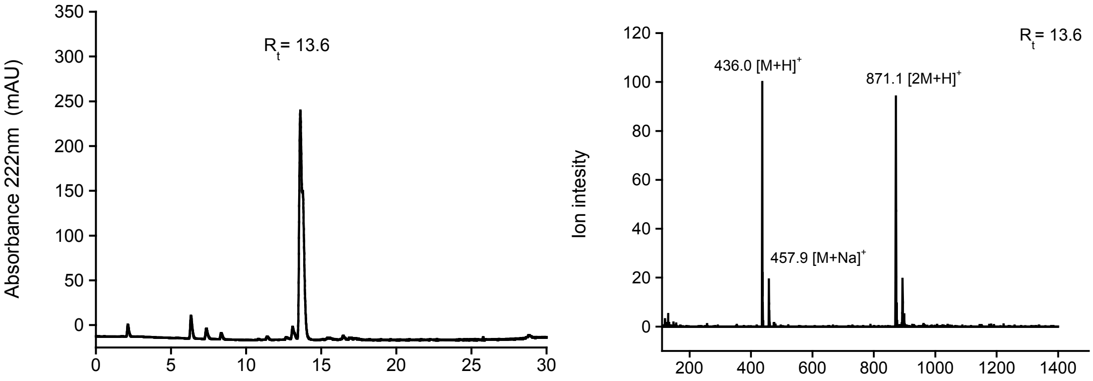

(2S)-N-((S,E)-5-phenyl-1-(phenylsulfonyl)pent-1-en-3-yl)piperidine-2-carboxamide Lm1msed42 (hPro-hPhe-VS)

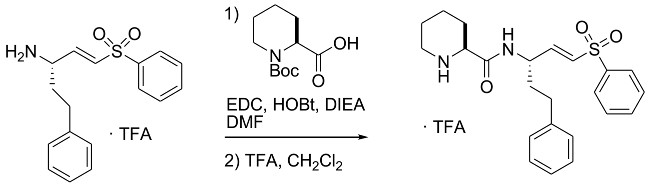

To a solution of (S)-N-Boc-Piperidine-2-carboxylic acid (211 mg, 0.92 mmol) in DMF (3 mL) was added EDC/HCl (177 mg, 0.92 mmol), HOBt (125 mg,0.92 mmol) and DIEA (0.6 mL, 3.1 mmol). After 10 min, (S)-3-benzenesulfonyl-1-phenethylallylamine trifluoroacetate (TFA•HphVSPh, X) (320 mg, 0.77 mmol) in DMF (3 mL) was added drop-wise. The reaction was stirred at rt for 12 h. The resulting solution was evaporated in vacuo to give a light yellow oil, which was diluted with aqueous NH_4Cl (10 wt%) and extracted with EtOAc (3 x 50 mL). The combined organic extracts were dried over Na_2SO_4, filtered and concentrated in vacuo. Purification by flash column chromatography (silica gel; using 40% EtOAc in hexanes) provided the protected product as a white foam.

To a solution of this intermediate in CH_2Cl_2 (5 mL) at 0 °C was added trifluoroacetic acid (5 mL). The solution was stirred at room temperature overnight, then evaporated to dryness in vacuo and finally and freeze-dried yielding a white solid that was identified as the trifluoroacetic salt of the desired product (216 mg, 0.41 mmol, 52%).

^{1}H NMR (*MeOD-d_4 δ*): 1.59-1.69 (m, 3H), 1.84-1.99 (m, 4H), 2.19 (d, J = 9.5 Hz, 1H),2.57-2.67 (m, 2H), 3.01 (t, J = 10.9 Hz, 1H), 3.38 (d, J = 12.6 Hz, 1H), 3.82 (dd, J = 11.1, 2.8 Hz, 1H), 4.57 (dd, J = 13.9, 5.7 Hz, 1H), 6.56 (d, J = 15.2 Hz, 1H), 6.91 (dd, J = 15.1, 5.9 Hz, 1H), 7.14-7.18 (m, 3H), 7.22-7.27 (m, 2H), 7.58-7.62 (m, 2H), 7.67-7.71 (m, 1H), 7.88 (d, J = 7.6 Hz, 2H).

^{13}C NMR (*MeOD-d_4 δ*): 22.8 (CH_2), 23.1 (CH_2), 28.7 (CH_2), 33.0 (CH_2), 36.2 (CH_2), 44.9 (CH_2), 50.8 (CH), 59.0 (CH), 127.3 (CH), 128.7 (CH), 129.5 (CH), 129.6 (CH), 130.7 (CH), 132.4 (CH), 134.9 (CH), 141.7 (C), 141.9 (C), 146.8 (CH), 169.8 (C).

ESI-MS: $[M+H]^+$ calcd. for $C_{23}H_{29}N_2O_3S$ = 413.1893 found 413.1896. (M.W. 526.5684)

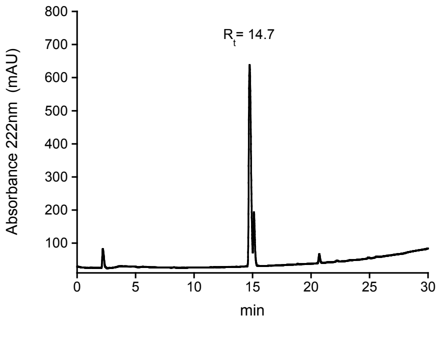

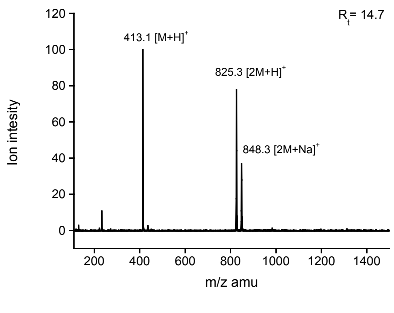

(2S)-2-amino-2-(2,3-dihydro-1H-inden-2-yl)-N-((S,E)-5-phenyl-1-(phenylsulfonyl)pent-1-en-3-yl)acetamide Lm1msed44 **(Igl-hPhe-VS)**

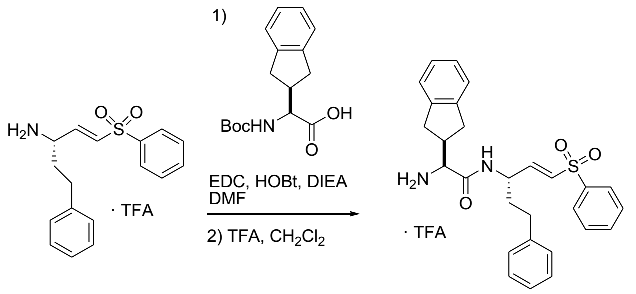

^{1}H NMR (*MeOD-d$_4$ δ*): 1.96 (td, *J* = 15.3, 7.5 Hz, 2H), 2.66 (dd, *J* = 15.6, 8.3 Hz, 2H), 2.88 (t, *J* = 5.60 Hz, 4H), 3.00-3.06 (m, 1H), 3.95 (dd, *J* = 5.4, 1.2 Hz, 1H), 4.60 (q, *J* = 6.8 Hz, 1H), 6.64 (d, *J* = 15.2 Hz, 1H), 6.89 (dd, *J* = 15.1, 6.6 Hz, 1H), 7.16 (bs, 7H), 7.24 (d, *J* = 7.1 Hz, 2H), 7.54-7.58 (m, 2H), 7.62-7.68 (m, 1H), 7.85 (d, *J* = 7.68 Hz, 2H).

^{13}C NMR (*MeOD-d$_4$ δ*): 32.9 (CH_2), 36.2 (CH_2), 36.3 (CH_2), 36.4 (CH_2), 43.1 (CH), 51.2 (CH), 57.8 (CH), 125.5 (CH), 127.3 (CH), 128.0 (CH), 128.7 (CH), 129.5 (CH), 129.6 (CH), 130.7 (CH), 133.2 (CH), 134.9 (CH), 141.6 (C), 142.0 (C), 142.1 (C), 142.2 (C), 146.0 (CH), 169.2 (C).

ESI-MS: $[M+H]^+$ calcd. for $C_{28}H_{31}N_2O_3S$ = 475.2050 found 475.2049. (M.W. 588.6377)

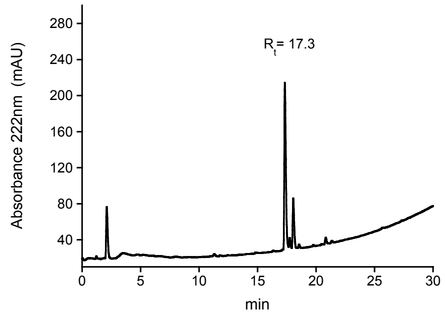

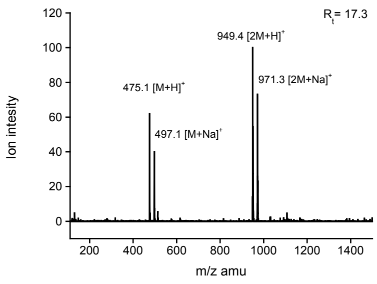

***tert*-Butyl (S)-5-(benzyloxy)-1-formylpentylcarbamate** Lm1msed46

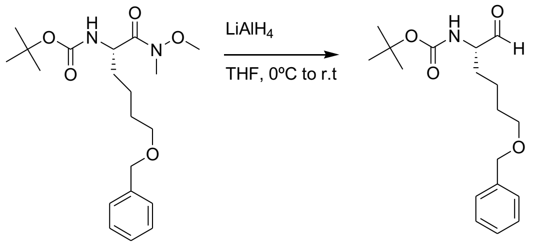

To a solution of *tert*-butyl (S)-1-(N-methoxy-N-methylcarbamoyl) -5-(benzyloxy) pentylcarbamate (2.2 g, 5.8 mmol) in dry THF (50 mL) at 0 °C was added $LiAlH_4$ (0.26 g, 7 mmol) over 10 min, with vigorous stirring. The mixture was stirred for an additional 20 min at 0 °C, whereupon cold water was carefully added until effervescence ceased. A cold HCl solution (1 M) was added to break up the gelatinous emulsion until pH 6~7. Upon dilution with H_2O (150 mL) and extraction with EtOAc (3 x 75 mL), the combined organic extracts dried over Na_2SO_4, filtered and concentrated in vacuo. Purification by flash column chromatography (silica gel; using 40% EtOAc in hexanes) provided the

product *tert*-Butyl (S)-5-(benzyloxy)-1-formylpentylcarbamate as a yellow oil (1.36 g, 4.23 mmol, 73%).

^{1}H NMR (*DCCl$_3$ δ*): 1.44 (s, 9H), 1.49-1.72 (m, 6H), 3.47 (t, *J* = 6.2 Hz, 2H), 4.21 (dd, *J* = 12.4, 6.3 Hz, 1H), 4.84 (s, 2H), 5.08 (d, *J* = 6.4 Hz, 1H), 7.32 (s, 5H), 9.56 (s, 1H).

^{13}C NMR (*DCCl$_3$*): 22.0 (CH_2), 28.2 (CH_3), 28.9 (CH_2), 29.3 (CH_2), 59.7 (CH), 69.7 (CH_2), 72.9 (CH_2), 80.0 (C), 127.5 (CH) 127.6 (CH), 128.3 (CH), 138.4 (C), 155.5 (C), 199.9 (CH).

ESI-MS: $[M+H]^+$ calcd. for $C_{18}H_{27}N_1O_4Na$ = 344.1832 found 344.1832. (M.W. 321.4113)

***tert*-Butyl (S,E)-7-(benzyloxy)-1-(phenylsulfonyl)hept-1-en-3-ylcarbamate** Lm1msed47

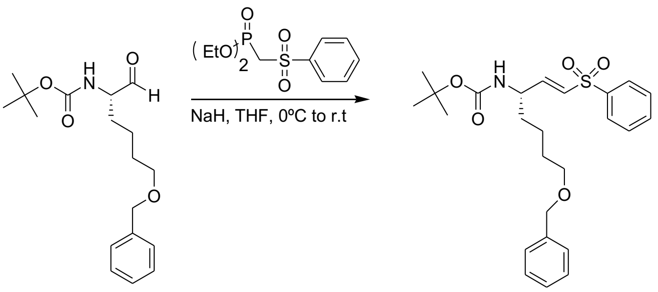

To a cooled (0°C) suspension of hexane-washed NaH (60% in mineral oil; 0.36 g, 9 mmol) in dry THF (50 mL) was added drop-wise diethyl[(benzenesulfonyl)methyl]phosphonate (2.4 g, 8.2 mmol) in dry THF (10 mL) via syringe. The mixture was stirred for an additional 30 min at 0 °C and *tert*-butyl (S)-5-(benzyloxy)-1-formylpentylcarbamate (2.30 g, 7.2 mmol) in dry THF (10 mL) was added drop-wise. The stirring was continued for 1 h, before a cold 10 wt% NH_4Cl solution was added to break up the gelatinous emulsion until pH 6~7. The solution was concentrated in vacuo, diluted with water (100 mL) and extracted with EtOAc (3 x 75 mL). The combined organic extracts were dried over Na_2SO_4, filtered and concentrated under vacuum. Purification by flash column chromatography (silica gel; using 60% EtOAc in hexanes) provided the product *tert*-Butyl (S,E)-7-(benzyloxy)-1-(phenylsulfonyl)hept-1-en-3-ylcarbamate (8) as a light yellow oil (2.74 g, 6.0 mmol, 83%).

^{1}H NMR (*DCCl$_3$ δ*): 1.37 (s, 9H), 1.42-1.46 (m, 4H), 1.58-1.61 (m, 2H), 3.44 (t, *J* = 6.1 Hz, 2H), 4.34 (bs, 1H), 4.47 (s, 2H), 4.57 (d, *J* = 8.4 Hz, 1H), 6.40 (dd, *J* = 15.0, 1.2 Hz, 1H), 6.87 (dd, *J* = 15.0, 4.7 Hz, 1H), 7.32 (bs, 5H), 7.48-7.63 (m, 3H), 7.86 (d, *J* = 7.2 Hz, 1H).

^{13}C NMR (*DCCl$_3$*): 22.4 (CH_2), 28.2 (CH_3), 29.2 (CH_2), 33.9 (CH_2), 50.9 (CH), 69.6 (CH_2), 72.9 (CH_2), 79.9 (C), 127.5 (CH), 127.6 (CH), 128.3 (CH), 129.2 (CH), 130.1 (CH), 133.4 (CH), 138.3 (C), 140.2 (C), 146.6 (CH), 154.8 (C).(*) Bidimensional experiments show that two CH share the same magnetic shift.

ESI-MS: $[M+H]^+$ calcd. for $C_{20}H_{32}N_2O_5Na$ = 482.1972 found 482.1972. (M.W. 459.5982)

(2S)-N-((S,E)-7-(benzyloxy)-1-(phenylsulfonyl)hept-1-en-3-yl)piperidine-2-carboxamide Lm1msed55 **(hPro-nLeu(O-Bzl)-VS)**

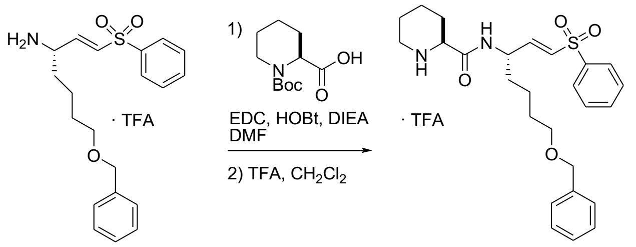

^{1}H NMR (*MeOD-d_4 δ*):1.35-1.45 (m, 2H) , 1.56-1.69 (m, 7H), 1.82-1.88 (m, 2H), .2.14 (d, J = 9.5 Hz, 1H), 2.97 (t, J = 12.4 Hz, 1H), 3.36 (d, J = 12.9 Hz, 1H), 3.45 (t, J = 6.3 Hz, 2H), 3.78 (dd, J = 11.2, 2.9 Hz, 1H), 4.46 (s, 2H), 4.56 (dd, J = 13.3, 5.9 Hz, 1H), 6.56 (d, J = 15.2 Hz, 1H), 6.89 (dd, J = 15.2, 5.7 Hz, 1H),7.30-7.33 (bs, 5H), 7.59 (t, J = 7.7 Hz, 2H), 7.68 (t, J = 7.4 Hz, 1H), 7.87 (d, J = 7.6 Hz, 2H).

^{13}C NMR (*MeOD-d_4 δ*): 22.7 (CH_2), 22.9 (CH_2), 23.6 (CH_2), 28.6 (CH_2), 30.0 (CH_2), 33.9 (CH_2), 44.9 (CH_2), 51.2 (CH), 58.9 (CH), 70.9 (CH_2), 73.8 (CH_2), 128.6 (CH), 128.7 (CH), 128.9 (CH), 129.4 (CH), 130.6 (CH), 132.0 (CH), 134.9 (CH), 139.7 (C), 141.5 (C), 147.2 (CH), 169.8 (C).

ESI-MS: $[M+H]^+$ calcd. for $C_{26}H_{35}N_2O_4S$ = 471.2312 found 471.2314. (M.W. 584.6481)

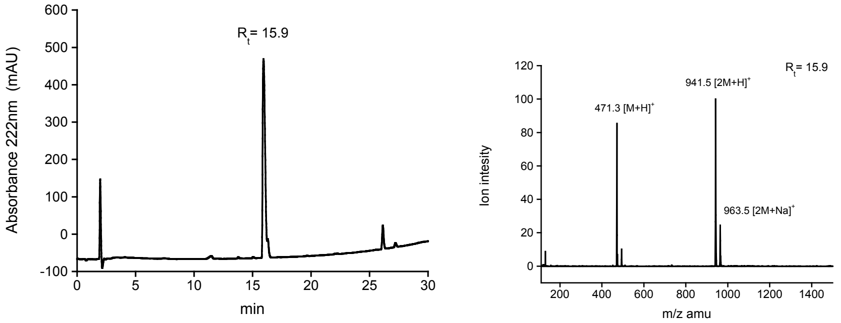

(2S)-2-amino-N-((S,E)-7-(benzyloxy)-1-(phenylsulfonyl)hept-1-en-3-yl)-3-(naphthalen-2-yl)propanamide Lm1msed56 **(2Nal-nLeu(O-Bzl)-VS)**

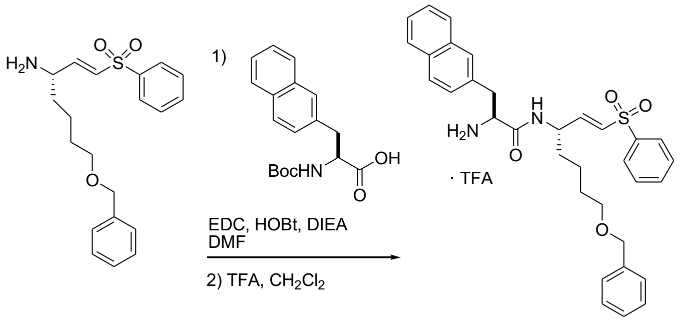

^{1}H NMR (*MeOD-d_4 δ*): 1.29-1.37 (m, 2H), 1.50-1.61 (m, 4H), 3.18-3.28 (m, 3H), 3.41 (t, J = 6.3 Hz, 1H), 4.15 (t, J = 7.3 Hz, 1H), 4.53 (q, J = 6.4 Hz, 1H), 4.40 (s, 2H), 6.27 (d, J = 15.2 Hz, 1H), 6.74 (dd, J = 15.2, 5.8 Hz, 1H), 7.30 (bs, 6H), 7.51 (bs, 4H), 7.63 (t, J = 7.4 Hz, 1H), 7.69-7.74 (m 3H), 7.83-7.87 (m, 3H).

^{13}C NMR (*MeOD-d_4 δ*): 23.5 (CH_2), 30.2 (CH_2), 34.1 (CH_2), 38.8 (CH_2), 51.4 (CH), 55.7 (CH), 70.9 (CH_2), 73.9 (CH_2), 127.3 (CH), 127.6 (CH), 128.1 (CH), 128.6 (CH), 128.7 (CH), 128.8 (CH), 128.9 (CH), 129.0 (CH), 129.4 (CH), 129.6 (CH), 130.1 (CH), 130.6 (CH), 132.2 (CH), 132.9 (C), 134.3 (C), 134.8 (CH), 135.0 (C), 139.7 (C), 141.6 (C), 146.8 (CH), 169.3 (C).

ESI-MS: $[M+H]^+$ calcd. for $C_{33}H_{37}N_2O_4S$ = 557.2469 found 557.2464. (M.W. 670.7383)

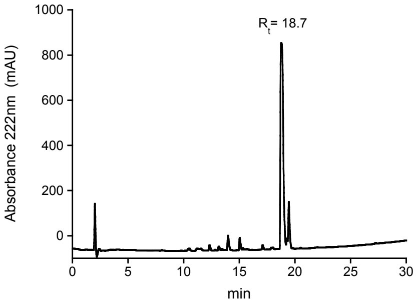

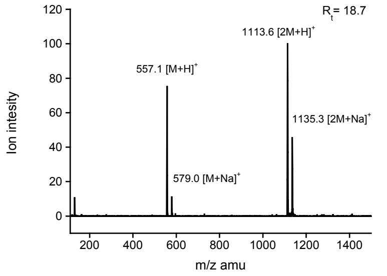

(2S)-2-amino-N-((S,E)-7-(benzyloxy)-1-(phenylsulfonyl)hept-1-en-3-yl)-2-(2,3-dihydro-1H-inden-2-yl)acetamide Lm1msed57 **(Igl-nLeu(O-Bzl)-VS)**

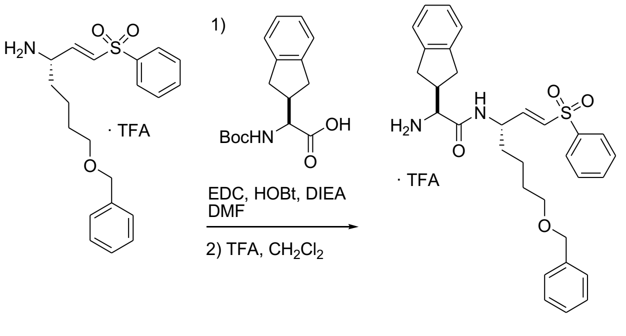

^{1}H NMR (*MeOD-d_4 δ*): 1.37-1.47 (m, 2H), 1.58-1.70 (m, 4H), 2.84 (dd, J = 18.7, 5.2 Hz, 4H), 2.99-3.04 (m, 1H), 3.47 (t, J = 6.2 Hz, 2H), 3.89 (d, J = 6.8 Hz, 1H), 4.47 (s, 2H), 4.58 (dd, J = 13.9, 6.8 Hz, 1H), 6.63 (d, J = 15.1 Hz, 1H), 6.86 (dd, J = 15.1, 6.5 Hz, 1H), 7.16 (bs, 4H), 7.32 (bs, 5H), 7.54 (t, J = 7.7 Hz, 2H), 7.60-7.67 (m, 1H), 7.84 (d, J = 7.8 Hz, 2H).

^{13}C NMR (*MeOD-d_4 δ*): 23.7 (CH_2), 30.2 (CH_2), 34.0 (CH_2), 36.3 (CH_2), 43.1 (CH), 51.5 (CH), 57.8 (CH), 71.0 (CH_2), 73.9 (CH_2), 125.5 (CH), 128.0 (CH), 128.6 (CH), 128.7 (CH), 128.9 (CH), 129.4 (CH), 130.7 (CH), 132.9 (CH), 134.9 (CH), 139.6 (C), 142.1 (C), 142.2 (C), 146.4 (CH), 169.2 (C).

ESI-MS: $[M+H]^+$ calcd. for $C_{31}H_{37}N_2O_4S$ = 533.2469 found 533.2463. (M.W. 646.7169)

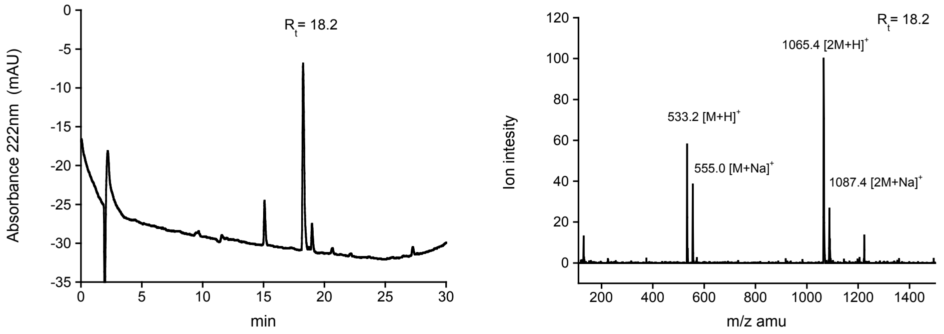

(2S)-2-amino-N-((S,E)-7-(benzyloxy)-1-(phenylsulfonyl)hept-1-en-3-yl)-3-(4-hydroxy-3-nitrophenyl)propanamide Lm1msed58 **(Tyr(NO2)-nLeu(O-Bzl)-VS)**

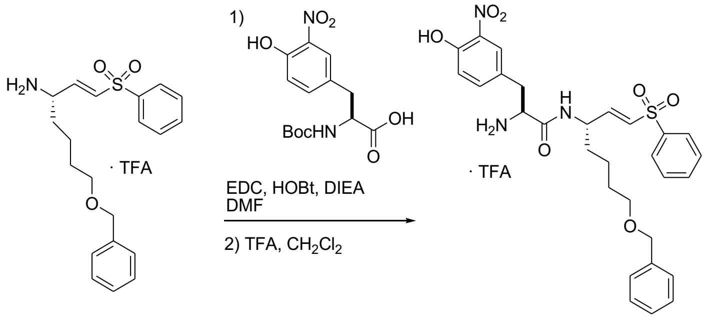

^{1}H NMR (*MeOD-d₄ δ*): 1.35-1.41 (m, 2H), 1.54-1.63 (m, 4H), 3.10 (dq, *J* = 14.1, 7.4 Hz, 2H), 3.45 (t, *J* = 6.2 Hz, 2H), 4.05 (t, *J* = 7.3 Hz, 1H), 4.45 (s, 2H), 4.55 (dd, *J* = 12.9, 6.3 Hz, 1H), 6.25 (d, *J* = 15.2 Hz, 1H), 6.72 (dd, *J* = 15.2, 5.8 Hz, 1H), 7.15 (d, *J* = 8.6 Hz, 1H), 7.30 (bs, 5H), 7.44 (dd, *J* = 8.6, 1.9 Hz, 1H), 7.58 (t, *J* = 7.6 Hz, 2H), 7.67 (t, *J* = 7.4 Hz, 1H), 7.85 (d, *J* = 7.6 Hz, 2H), 7.99 (d, *J* = 1.8 Hz, 1H).

^{13}C NMR (*MeOD-d₄ δ*): 23.5 (CH_2), 30.2 (CH_2), 34.2 (CH_2), 37.3 (CH_2), 51.3 (CH), 55.3 (CH), 70.9 (CH_2), 73.9 (CH_2), 121.8 (CH), 127.0 (CH), 127.4 (CH), 128.7 (CH), 128.8 (CH), 128.9 (CH), 129.4 (CH), 130.6 (C), 132.3 (CH), 134.9 (CH), 135.9 (C), 138.9 (CH), 139.7 (C), 141.6 (C), 146.7 (CH), 155.1 (C), 168.8 (C).

ESI-MS: $[M+H]^+$ calcd. for $C_{29}H_{34}N_3O_7S$ = 568.2112 found 568.2102. (M.W. 681.6766).

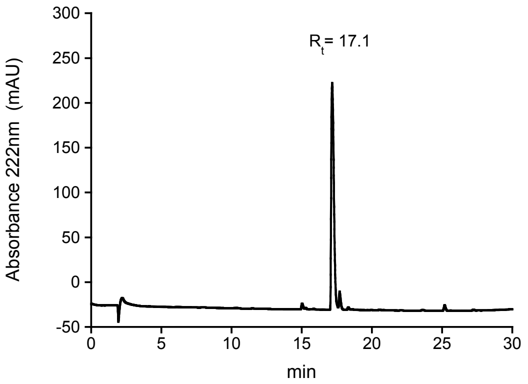

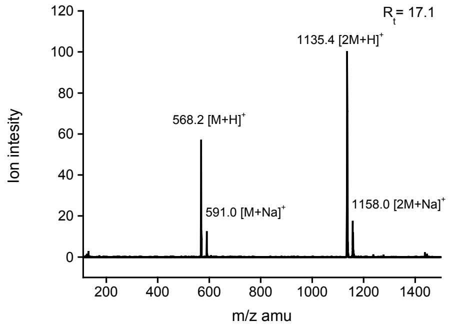

(2S)-2-amino-N-((S,E)-7-(benzyloxy)-1-(phenylsulfonyl)hept-1-en-3-yl)-3-(1H-indol-3-yl)propanamide Lm1msed68 **(Trp-nLeu(O-Bzl)-VS)**

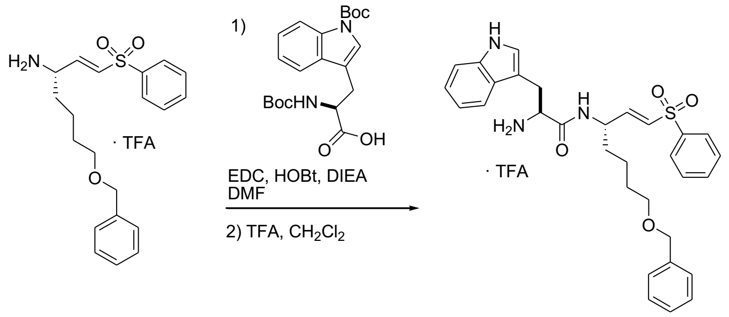

^{1}H NMR (*MeOD-d_4 δ*): 1.31-1.37 (m, 2H), 1.49-1.59 (m, 4H), 3.20 (dd, J = 14.5, 7.0 Hz, 1H), 3.34 (d, J = 7.8 Hz, 1H), 3.43 (t, J = 6.2 Hz, 2H), 4.09 (t, J = 7.4 Hz, 1H),4.44 (s, 2H), 4.52 (q, J = 6.2 Hz, 1H), 6.12 (d, J = 15.2 Hz, 1H), 6.73 (dd, J = 15.2, 5.5 Hz, 1H), 7.05 (t, J = 7.4 Hz, 1H), 7.21 (bs, 2H), 7.30 (bs, 5H), 7.40 (d, J = 8.1 Hz, 1H), 7.54 (t, J = 7.6 Hz, 2H), 7.59-7.66 (m, 2H), 7.80 (d, J = 7.8 Hz, 2H).

^{13}C NMR (*MeOD-d_4 δ*): 23.5 (CH_2), 29.0 (CH_2), 30.2 (CH_2), 34.2 (CH_2), 51.2 (CH), 54.9 (CH), 71.0 (CH_2), 73.9 (CH_2),108.0 (C), 112.9 (CH), 119.1 (CH), 120.4 (CH), 123.0 (CH), 125.5 (CH), 128.1 (C), 128.7 (CH), 128.8 (C), 128.9 (CH), 129.4 (CH), 130.6 (CH), 132.0 (CH), 134.8 (CH), 138.2 (C), 139.7 (C), 141.6 (C), 146.8 (CH), 169.8 (C).

ESI-MS: $[M+H]^+$ calcd. for $C_{31}H_{36}N_3O_4S$ = 546.2421 found 546.2438. (M.W. 773.7390)

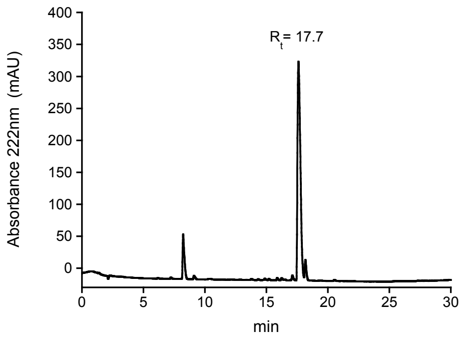

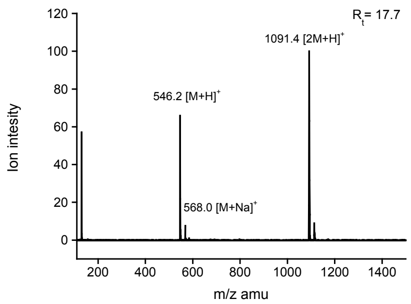

(S,E)-7-(benzyloxy)-1-(phenylsulfonyl)hept-1-en-3-amine trifluoroacetate Lm1msed69

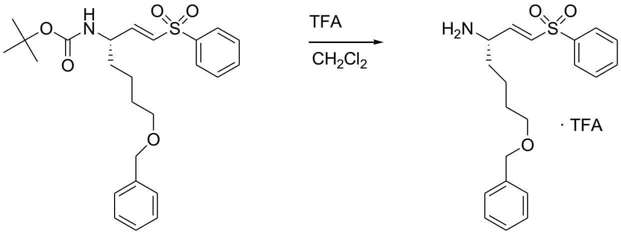

To a cooled (0°C) solution of *tert*-butyl (S,E)-7-(benzyloxy)-1-(phenylsulfonyl)hept-1-en-3-ylcarbamate (0.2 g, 0.43 mmol) in CH_2Cl_2 (5 mL) was added drop-wise TFA (1 mL) via syringe and stirred for 30 min. After checking by HPLC-MS that all the starting material was consumed, the solvent was removed under reduced pressure, and the residual TFA was removed by co-distillation with CH_2Cl_2 and finally dried in vacuo to give (S,E)-7-(benzyloxy)-1-(phenylsulfonyl)hept-1-en-3-amine as a trifluoroacetic salt (0.2 g; 0.43 mmol, 99%). This material was pure enough to be used in the next step without further purification.

^{1}H NMR (***MeOD$_4$ δ***): 1.35-1.44 (m, 2H), 1.55-1.66 (m, 2H), 1.71-1.83 (m, 2H), 3.45 (t, J = 6.0 Hz, 2H), 3.98 (dd, J = 13.9, 6.9 Hz, 1H), 4.46 (s, 2H), 6.86 (dd, J = 15.3, 6.9 Hz, 1H), 6.97 (d, J = 15.3 Hz, 1H), 7.32 (bs, 5H), 7.56-7.62 (m, 2H), 7.69 (d, J = 7.1 Hz, 1H), 7.91 (d, J = 7.1 Hz, 2H).

^{13}C NMR (***MeOD$_4$***): 22.8 (CH_2), 29.7 (CH_2), 32.9 (CH_2), 52.2 (CH), 70.4 (CH_2), 73.6 (CH_2), 128.5 (CH), 128.6 (CH), 128.7 (CH), 129.2 (CH), 130.5 (CH), 134.9 (CH), 136.2 (CH), 139.4 (C), 140.6 (C), 140.8 (CH), 161 (q, C, TFA).

ESI-MS: $[M+H]^+$ calcd. for $C_{20}H_{25}NO_3SNa$ = 382.1447 found 382.1458. (M.W. 473.5057)

(2R)-2-amino-N-((S,E)-7-(benzyloxy)-1-(phenylsulfonyl)hept-1-en-3-yl)-3-(1H-indol-3-yl)propanamide Lm1msed71 (***L*-Trp-nLeu(O-Bzl)-VS)**

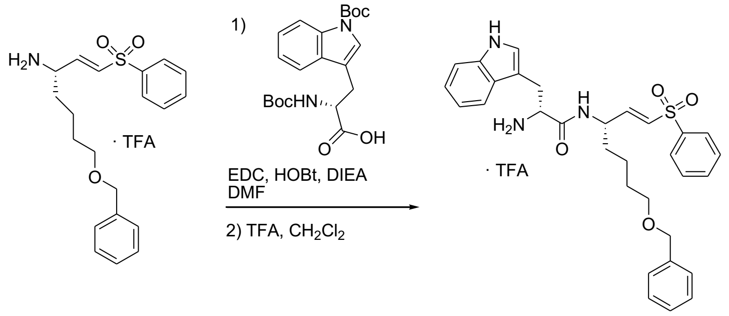

^{1}H NMR (*MeOD-d₄ δ*): 0.95-1.02 (m, 2H), 1.09-1.18 (m, 1H), 1.28-1.35 (m, 1H), 1.37-1.45 (m, 2H), 3.21 (dd, *J* = 14.4, 7.2 Hz, 1H), 3.33-3.38 (m, 3H), 4.05 (t, *J* = 7.6 Hz, 1H), 4.43 (bs, 3H), 6.61 (d, *J* = 15.2 Hz, 1H), 6.81 (dd, *J* = 15.1, 5.2 Hz, 1H), 7.04 (t, *J* = 7.4 Hz, 1H), 7.11 (t, *J* = 7.5 Hz, 1H), 7.16 (s, 1H), 7.28 (s, 2H), 7.29 (s, 2H), 7.35 (d, *J* = 8.1 Hz, 1H), 7.54-7.59 (m, 4H), 7.66 (t, *J* = 7.4 Hz, 1H), 7.84 (d, *J* = 7.5 Hz, 2H).

^{13}C NMR (*MeOD-d₄ δ*): 23.2 (CH_2), 29.0 (CH_2), 30.1 (CH_2) 33.9 (CH_2), 51.3 (CH), 55.3 (CH), 70.9 (CH_2), 73.9 (CH_2), 108.2 (C), 112.7 (CH), 119.1 (CH), 120.3 (CH) 122.9 (CH), 125.5 (CH), 128.4 (C), 128.7 (CH), 128.8 (CH), 129.4 (CH), 130.6 (CH), 132.0 (CH), 134.8 (CH), 138.2 (C), 139.8 (C), 141.7 (C), 147.0 (CH), 169.8 (C).

ESI-MS: $[M+H]^+$ calcd. for $C_{31}H_{36}N_3O_4S$ = 546.2421 found 546.2411. (M.W. 773.7390)

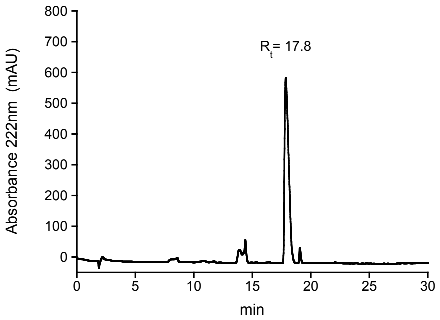

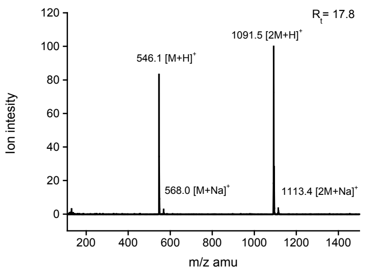

Supplement: Supplementary file 1 — Fig. S1. Michaelis–Menten fits for DPAP3. Fig. S2. Representative irreversible inhibition fits. Fig. S3. Michaelis–Menten fits for CatC. Appendix S1. Synthesis and characterization of inhibitors and substrates. [file FEBS-286-3998-s001.zip › febs14953-sup-0001-Supinfo.pdf]
